# A Framework for Identifying Plant Species to Be Used as ‘Ecological Engineers’ for Fixing Soil on Unstable Slopes

**DOI:** 10.1371/journal.pone.0095876

**Published:** 2014-08-08

**Authors:** Murielle Ghestem, Kunfang Cao, Wenzhang Ma, Nick Rowe, Raphaëlle Leclerc, Clément Gadenne, Alexia Stokes

**Affiliations:** 1 AgroParis Tech, UMR AMAP, Montpellier, France; 2 Xishuangbanna Tropical Botanical Garden, Chinese Academy of Sciences, Menglun, Mengla, Yunnan, China; 3 Kunming Institute of Botany, Kunming, Yunnan, China; 4 CNRS, UMR AMAP, Montpellier, France; 5 CNRS, UMR CEFE, Montpellier, France; 6 AgroParisTech, Centre de Nancy, Nancy, France; 7 INRA, UMR AMAP, Montpellier, France; Centro de Investigacion Cientifica y Educacion Superior de Ensenada, Mexico

## Abstract

Major reforestation programs have been initiated on hillsides prone to erosion and landslides in China, but no framework exists to guide managers in the choice of plant species. We developed such a framework based on the suitability of given plant traits for fixing soil on steep slopes in western Yunnan, China. We examined the utility of 55 native and exotic species with regard to the services they provided. We then chose nine species differing in life form. Plant root system architecture, root mechanical and physiological traits were then measured at two adjacent field sites. One site was highly unstable, with severe soil slippage and erosion. The second site had been replanted 8 years previously and appeared to be physically stable. How root traits differed between sites, season, depth in soil and distance from the plant stem were determined. Root system morphology was analysed by considering architectural traits (root angle, depth, diameter and volume) both up- and downslope. Significant differences between all factors were found, depending on species. We estimated the most useful architectural and mechanical traits for physically fixing soil in place. We then combined these results with those concerning root physiological traits, which were used as a proxy for root metabolic activity. Scores were assigned to each species based on traits. No one species possessed a suite of highly desirable traits, therefore mixtures of species should be used on vulnerable slopes. We also propose a conceptual model describing how to position plants on an unstable site, based on root system traits.

## Introduction

Eco-engineering has been defined as the long-term, ecological strategy to manage a site with regard to natural or man-made hazards [1]. Vegetation has long been recognized as useful for increasing slope stability with regard to shallow landslides and erosion [2]. Plant root systems have predominant effects on slope stability, compared to aboveground attributes [3]. To improve slope stability on a large scale, managers could focus initially on relatively small areas, or ‘hotspots’ [4]. With regard to soil erosion, these areas are defined as sites with soil erosion rates well above soil loss tolerance levels [5]. Hotspots often only occupy a small fraction of a catchment's area, but may be held responsible for a very significant contribution to overall sediment production, thus leading to off-site problems [6]. Reducing erosion or soil slippage on these degradation hotspots via an appropriate species or mixture of species would be an economic and efficient method to protect against large-scale landslides. Therefore, the appropriate root characteristics for fixing soil in hotspots should be identified. The optimum spatial positions of species within a hotspot could also be determined, depending on local soil and climatic conditions.

If plants could be used as ‘ecological engineers,’ i.e. can be used to create a sustainable ecosystem that adds value to both the environment and society [7], the most preferred species for slope stabilization would be native with local economic value. Individuals should possess root traits known to improve slope stability, where a trait is a well-defined, measurable property of an organism, usually measured at the individual level and used comparatively across species [8]. Lists of desirable root system traits for fixing soil on slopes are available [9], [10], [11] (Table 1). Individuals should possess extensive and deep root systems with strong and fast-growing roots that are slow to decompose [10]. However, the most commonly used traits for quantifying the contribution of plant root systems to slope stability are root area ratio (RAR), which is the surface area of roots over a given area of soil, and root maximal tensile strength. These traits are used to calculate the root mechanical contribution as additional soil cohesion [12], [13], [14], [15]. Nevertheless, it is recognised that these simple traits cannot describe the full root reinforcement mechanism on slopes, and that we should consider more complex suites of traits [11]. With regard to root mechanical traits, it has recently been shown that the mechanical behaviour of a root during a deformation, and not only its maximal tensile strength at breakage, should be used to calculate more realistic values of additional cohesion [16], [17], [18]. To improve slope stability, roots with a high tensile strength at breakage and a small tensile strain at breakage would be most efficient, because soil can bear only a very small displacement before it ruptures [11], [19], [20]. Strain in a root held in tension is composed of three phases: (i) an initial phase of stretching followed by (ii) a phase of reversible deformation called the elastic phase, which is sometimes, but not always, followed by (iii) a phase of non-linear and irreversible deformation called the plastic phase leading to breakage. To improve slope stability, the irreversible phase of deformation should be as small as possible because roots which can be re-mobilized will be more efficient in the long-term. Ghestem [21] showed that the maximal tensile strength required to cause root failure is correlated with the elastic characteristics (elastic strength, elastic strain and tensile modulus of elasticity) and thus represents the capacity of a root to deform reversibly. The ultimate tensile strain is correlated with the plastic characteristic of roots (plastic strain and plastic modulus) and thus represents the irreversible deformation of a root. Very few studies have examined such detailed root mechanical traits in the context of slope stability analyses [22], [23]. In most previous studies, where the contribution of vegetation to slope stability was determined, only the tensile strength of thin and fine roots was determined, and found to be correlated with cellulose and lignin content [24], [25]. Yet, thicker roots act as soil nails, pinning the root systems into the substrate. Therefore, [26] stipulated the importance of also measuring the bending resistance of thick roots, so that they could be incorporated into slope stability models [27].

**Table 1:** Desirable traits to compare species efficiency for stabilizing slopes and abbreviations used in the text.

Plant properties	Desirable traits	Abbreviations
Root abundance in soil	High number of plant stems per m^2^ of degraded slope	Nb stems.m-^2^
	High individual soil volume standardized by the collar diameter of the plant	ISV/Dc
	High root area ratio	RAR
Root mechanical resistance	High maximal tensile strength x high proportion of fine roots	T_max_ x RAR_f_
	Short ultimate tensile strain	ε_ult_
	High bending rigidity x high proportion of coarse roots	*EI* x RAR_c_
Root metabolic activity	Short ultimate tensile strain, proxy for high reversibility in tension	ε_ult_
	High root nitrogen content, proxy for high metabolic activity	N
	High cellulose content, proxy for long lifespan	CELL

Ultimate tensile strain is a trait representing both a mechanical property and a proxy for a physiological property: the ability of a root to recover after a deformation (reversible deformation).

Root architectural traits allow for the description of root system morphology and topology, each of which influence slope stability [11], [28]. The individual soil volume (ISV) is the root system's overall envelope, given by its maximum radius (horizontal extension) and its maximum depth (vertical extension) [29], [30], [31], and thus quantifies root spread of an individual on a slope.

Root physiological traits provide useful information about root system functioning and uptake capacity. Root metabolic activity (respiration rate and nutrient uptake) is correlated with root nitrogen (N) concentration; root longevity is correlated with root cellulose content [24], [32]. The ultimate tensile strain, correlated with the irreversible deformation of a root as described hereabove, also worth considering as root physiological trait: a short ultimate tensile strain means that the root is able to recover promptly after deformation without structural damage.

To determine the plant species useful for engineering slope stability, we analysed traits of plants growing along a degraded slope in southern China. The number of shallow landslides in China has increased enormously over the last 50 years, due mainly to deforestation, infrastructure and road construction [1], [33], [34]. The Chinese government has therefore launched a major landslide inventory named “monitoring and preventing of landslides by the masses” in 1990 [33] and two afforestation programs: the Natural Forest Protection Program (NFPP) in 1998 and the Sloping Land Conversion Programme in 1999 (SLCP, or “Grain for Green programme,” which aims at planting trees on existing agricultural land, concentrating on zones where slopes are >25°) [1], [35]. The results of these two programs are contrasted [35], [36], [37]. Case studies recorded high seedling mortality where tree species used for replanting were not suitable for the local environment [38], [39], degradation of local population welfare [40], more superficial erosion [41] and increased slope instability [42]. Within such a major socio-economic context, information on how species can be used to engineer slope stability is vital, especially during the early years after plantation on a bare slope, when the window of landslide susceptibility is greatest [43].

We discuss therefore, how plants can be screened for use as ‘ecological engineers’, through a better understanding of mechanical, architectural and physiological traits of their root systems. Results are discussed with regard to the optimisation of species mixtures and planting patterns on degraded slopes. As developed by [44] and [45] studying soil and gully erosion in Ethiopia, we aimed at producing a generic framework which can be used by managers to determine suites of plant traits useful for engineering slope stability with regard to shallow landslides.

## Material and Methods

### Ethics statement

Access to the field site was obtained from the Daxingdi forest service and Town Hall. Field sites were publicly owned and not protected. No protected species were sampled during this study.

### Study site

**Figure 1:**
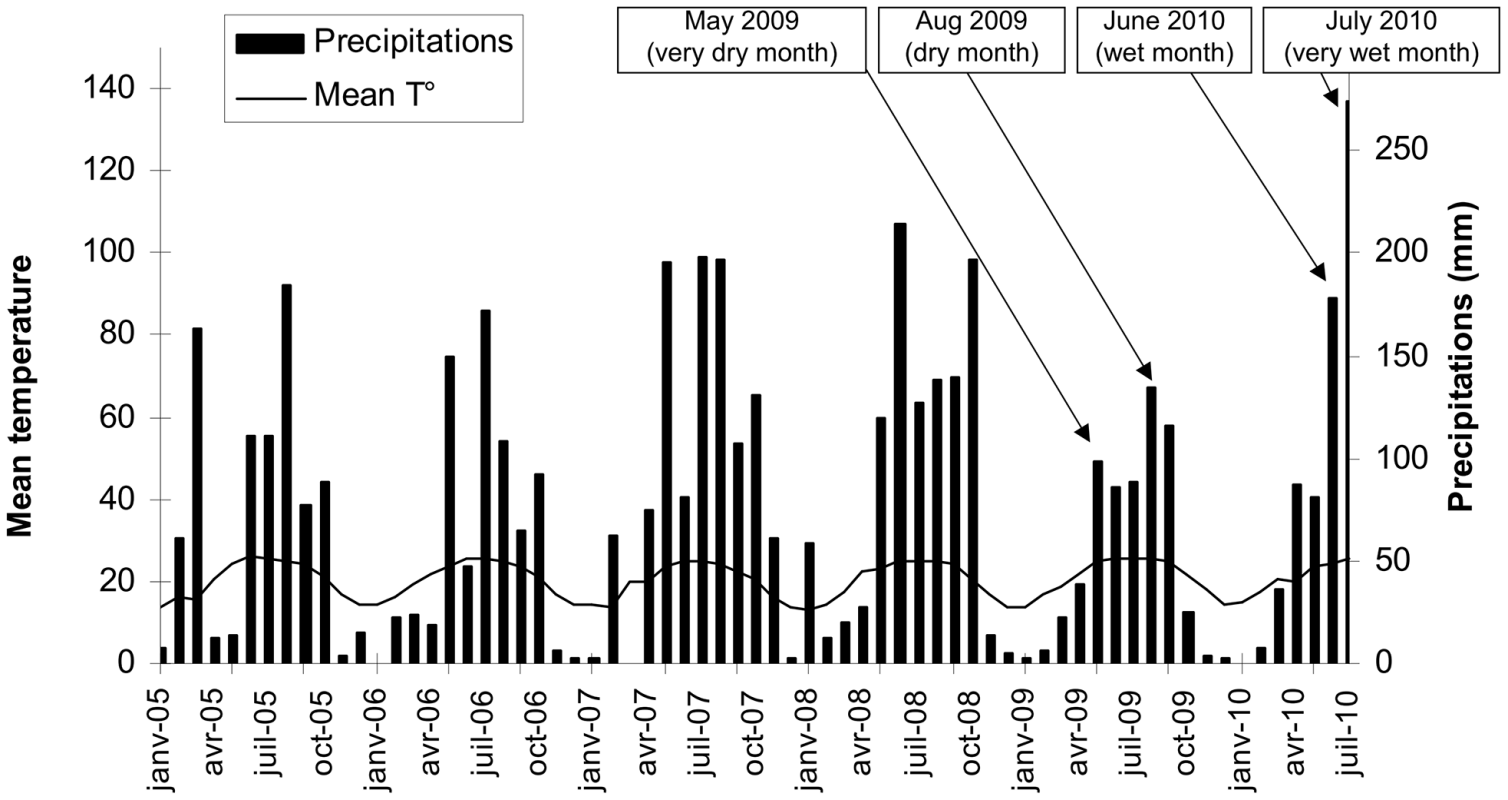
Mean temperature and precipitations in Liuku. Diagram from Liuku meteorological station situated 30 km south from the field site (source: Meteorological Bureau of Yunnan Province). Data from January 2005 to May 2010. Arrows show the months when roots were collected.

We studied root morphological, mechanical and physiological traits for species growing in the Yunnan province, southern China, where erosion and landslides are severe [46]. The study area (26°01′N, 98°50′E) was located near Daxingdi village, north of Liuku town in the Salween river valley. This part of China is under the influence of the Indian monsoon, and described as a “warm-dry climate”, being a combination of subtropical and alpine climates. Annual mean temperature (from 1961 to 2002) is 15.2°C, and mean annual precipitation is 1200 mm, the majority of which falls between May and October [14], [41]. Numerous landslides occur during the monsoon season (May-October) and soil erosion is severe, largely due to the cutting of roads through the steep slopes [1], [34]. The use of *Agava americana* L. to fix soil on steep slopes after road building is common practice in the region. We carried out fieldwork in 2009 and 2010. In 2010, precipitation during the summer months was particularly high (Figure 1) and shallow landslides throughout the area were numerous. At our study sites, corn (*Zea mays* L.) was cultivated from 1980, after deforestation, until 1999 when the SLCP was initiated. Several species of trees and shrubs were then planted, including *Agava americana* L., *Jatropha curcas* L., *Pueraria stricta* Kurz., *Ricinus communis* L., and *Vernicia fordii* Helmsl.

We identified two study sites. One site was an active shallow landslide approximately 30 m wide and 50 m long. The origin of the landslide may have been due to severe erosion leading to gully formation and eventual soil slippage. As soil slippage at this site was active, we considered it as a degradation hotspot. The second site was located at 3 m from the first site. A shallow landslide had occurred at the second site in 2000, after an extreme precipitation event. The type of soil slippage observed was typical of western Yunnan [34]. The specific vegetation composition and distribution was similar to other sites along the Salween river valley (data not shown). Since 2000, species have colonized this second site naturally or have been planted within the SLCP, therefore we presumed that it was relatively stable. The two sites were located at an altitude of 1010 m. Slope angle was 35–45° at the degradation hotspot and 50–60° at the stable site. Both sites were oriented at 300° from due north.

### Soil characteristics

#### Soil profiles

**Figure 2:**
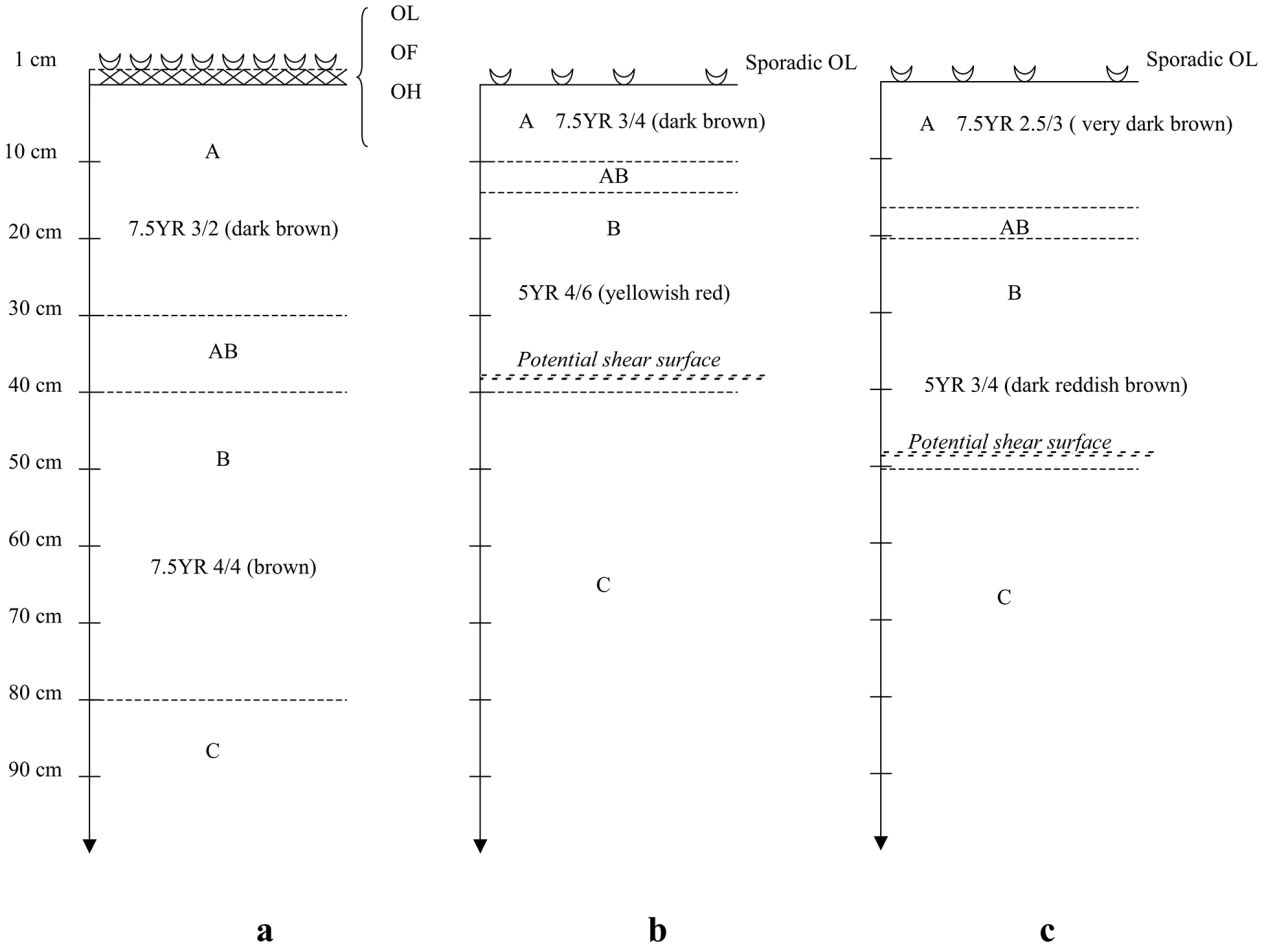
Soil profiles at the study site. Soil horizons at a) a reference site with no previous evidence of landslides or erosion; b) the stable site where a shallow landslide had occurred eight years previously and c) the hotspot. Colours were identified using a Munsell colour chart (Munsell 1947). OL: fresh litter, OF: fermenting litter, OH: litter with humic substances and well-transformed organic matter, A: organico-mineral layer, AB: mixture between A and B, B: layer of bedrock alteration, pieces of bedrock are visible, C: bedrock, mineral layer (Legros 2000; Baize and Girard 1995).

We determined soil profiles at the hotspot, stable site and also at a third site taken as reference. This reference site was situated 200 m from our sites at same altitude, covered with the same type of vegetation and was used for comparison of soil profiles (Figure 2). This reference site was considered highly stable because no evidence of previous landslides or erosion was found. Soil profiles to a depth of 1.0 m were examined at each site and described using colour charts [47]. Potential shear surfaces were identified as the limit between soil and bedrock horizons as is generally observed in the field and especially where percolating water stagnates [48], [49]. The soil was represented by a ferrallitic red carbonated soil with many mineral coloured spots, e.g. iron and manganese. In the third reference site with no previous evidence of a landslide, humus thickness was <1 cm, soil thickness was 0.7–2.0 m and the source rock was limestone. Humus was classified as a mesomull [50]. Source rock (limestone) emergence occurred at 0.5 m at the hotspot and at 0.4 m on the relatively stable site where a landslide had occurred 8 years previously (Figure 2).

#### Soil texture and chemical characteristics

Soil texture and chemical characteristics were measured at the hotspot and stable site. Samples were taken randomly throughout each plot from representative typical A- and at B-horizons i.e. n = 8 samples at 0.05 m and n = 8 samples at 0.35 m. Soil analyses were carried out on soil fractions finer than 2 mm. Sand (2.00–0.05 mm), silt (0.050–0.002 mm), and clay (<0.002 mm) contents were determined using the sedimentation and sieving method (Table 2), NF P 94–056 and 94–057 [51]. Soil pH was measured using a potentiometry method (LY/T1239–1999) [52], cation exchange capacity (CEC) was measured using the distillation method (LY/T1243–1999) and soil organic carbon and total nitrogen (N) contents were measured using a C-N analyzer (Thermo-Finnigan CHN analyser). The two sites possessed similar basic pH and CEC values (Table 2). CEC was high compared to typical values of soils containing 50% clay [53], indicating a high potential fertility. Soil organic carbon and total N concentrations (Table 2) were similar to those found in forest or pasture soils [53]. Soil organic carbon, representing soil organic matter (Soil organic matter = Soil organic carbon*1.7) [53], was more abundant at the hotspot compared to the stable site. The proportion of organic carbon on total N was higher at the hotspot compared to the stable site, indicating that mineralisation of organic matter was slower at the hotspot.

**Table 2:** Soil textural, chemical and physical characteristics in horizons A and B at the hotspot and stable site.

Soil property	Soil horizon	Stable site	Unstable hotspot
		n = 8	n = 8
Clay (%)	A	41.70 ± 6.73	49.21 ± 2.66
Silt (%)		44.04 ± 4.46	41.12 ± 4.25
Sand (%)		14.27 ± 1.69	9.68 ± 3.61
Clay (%)	B	47.62 ± 8.52	46.71 ± 11.49
Silt (%)		42.20 ± 6.59	39.03 ± 8.37
Sand (%)		10.18 ± 2.41	14.26 ± 7.34
		n = 8	n = 8
pH		8.42	8.33
CEC cmol(+).kg^-1^		51.51	43.81
Organic carbon (g.kg^-1^)	A	12.31	25.8 ± 1.9
	B		28.7 ± 1.4
Total nitrogen (g.kg^-1^)	A	0.91	1.5 ± 0.1
	B		0.7 ± 0.8
		n = 7	n = 7
Soil water content (%)in August 2009		27 ± 8.23	28 ± 6.62
Soil water content (%) in July 2010	A	18.28 ± 3.73	23.29 ± 8.28
	B	17.48 ± 3.26	15.3 ± 3.50
Soil water content (%)		60.0 ± 7.44	70.0 ± 3.00
Dry bulk density (g.cm^-3^)	A	0.88 ± 0.23	0.78 ± 0.03
	B	1.01 ± 0.18	0.99 ± 0.16
		n = 20	n = 20
Resistance with manual shear tore vane (kPa)		0.27 ± 0.02	0.17 ± 0.02***
Resistance with manual penetrometer (kPa)		3.25 ± 0.20	1.48 ± 0.13***
		n = 8	n = 8
Soil cohesion (kPa)		5.35 ± 1.97	0.49 ± 0.92
Angle of internal friction (°)		20. 21 ± 3.02	26.98 ± 1.41

The number (n) of samples is indicated for each test. When n<10, no statistical analyses were carried out. Data are means ± standard error.

***: Differences were significant at >0.001 level.

#### Soil physical characteristics

A manual shear tore vane (IMG I01 025) was used to estimate soil shear strength in situ at a depth of 0.20 m at 20 randomly located positions within each plot (Table 2). A manual penetrometer (Elmeg PEN-3960) was also used to estimate penetration at a depth of 0.20 m at 20 locations within each plot. Strain-controlled direct shear tests were carried out on eight reconstituted, drained 60 mm×60 mm×20 mm soil samples from each site and each horizon. Roots were removed during the reconstitution process [41]. Samples were not saturated prior to testing, and as they were kept sealed at 4°C after removal from the field, it can be assumed that soil moisture content was similar to that in field conditions. Samples were placed in a shear testing device (VJTech 2760A) and normal loads of 200, 300 and 500 N were applied as weights on three separate samples taken from the same block of soil [54]. A lateral displacement was applied at a rate of 0.8 mm min^−1^ until failure occurred and the peak shear force recorded. Soil cohesion and the angle of internal friction were obtained by the Mohr-Coulomb theory [54]. To obtain soil initial moisture content (*w_i_*), soil moisture content at saturation (*w_s_*) and soil dry bulk density (*ρ_d_*), a modified method from [53] was used on seven samples at the two sites (in 2009) and at A and B horizons (in 2010). First, samples were weighed while they were fresh (*m_i_*, initial mass). Samples were then dipped in paraffin and the volume of water occupied by the sample measured in a graduated cylinder (*v*, soil volume). Samples were then saturated with water and weighed (*m_s_*, saturated mass), before drying at 105°C until constant weight (*m_d_*, dry mass). 
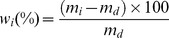
(Eq. 1)

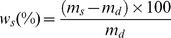
(Eq. 2)

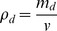
(Eq. 3)


### Choice of species

A species inventory was carried out in 2008 and 60 species identified (Species List S1). Before we selected species for the study, we obtained information on species' ecology and economic value from the literature, field observations and discussions with local people. We then screened for two necessary criteria: the species must be present on disturbed sites in the region and must be compatible with other species, i.e. not be invasive. Amongst species answering to those two criteria, ecological characteristics were considered e.g. plant lifespan (annual, biannual, perennial), rooting type (through visual observations) and growth form (herb, shrub, climber, creeping plant, tree). Any economic and ethnobotanical properties were noted e.g. food and fodder use, medicinal purposes, fertilizer, fuel or handcrafts. Species were then classified according to these properties (Species List S1). From this initial screening, we selected nine species for this study: *Agava americana*, *Arthraxon hispidus* (Thunb. ex Murray) Makino, *Artemisia codonocephala* DC., *Bauhinia championii* Benth., *Chloris anomala* B. S. Sun & Z. H. Hu, *Ficus tikoua* Bureau, *Jatropha curcas*, *Pueraria stricta*, *Rhus chinensis* Miller. All species were pioneers. They were present at both sites at the beginning of the rainy season, when slopes are more prone to shallow landslides and water erosion. Species were not invasive and were of economic value for the local population. Five different growth forms were also represented i.e. herb, shrub, creeper, climber, and tree forms.

### Plant abundance

In August 2009 and July 2010, the density of each of the nine species was measured at both sites in two transects along the slope. Eight 1 m^2^ quadrats were placed every 4 m along each transect. Within each quadrat, we counted the number of individuals of each of the nine species and the percentage of soil covered by the vertical projection of their canopy at a height of 30 cm above the soil surface.

### Choice of individuals

**Figure 3:**
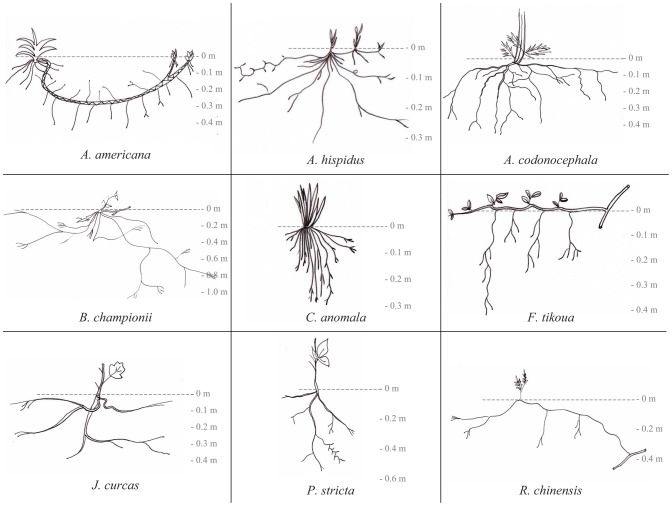
Description of the root systems of the nine studied species. Root systems of the nine species and their rooting depth (m). *A. americana*'s root system was composed of an underground stem from which emerged thin roots; *A. hispidus*'s root system comprised only a few thin roots emerging from the plant collar; *A. codonocephala* possessed a root system with long lateral roots turning downwards over time; *B. championii* had long and deep roots, which deviated on encountering an obstacle and which were densely branched; *C. anomala* possessed a tufted and shallow root system; vertical roots of *F. tikoua* emerged from creeping stems; *J. curcas* and *P. stricta* both possessed taproot systems, but roots of *P. stricta* were deeper and more densely branched; *R. chinensis* had a sprouting root system comprising long, deep and scarcely branched roots. Note that the scale for soil depth (y axis) differs between species for easier viewing.

For each of the nine species, 12 individuals were chosen i.e. six at each site. All individuals were chosen within the same size range. Plant height and stem basal diameter were measured (Table 3), but are not indicative of age because of occasional cattle grazing. By comparing these individuals with reference plants that germinated during the 3 years we worked at the site, we estimated that the individuals we studied were 3–6 years old. Root systems were excavated by hand (Figure 3). Excavations were carried out with extreme caution, so as to not damage root systems (Table 3).

**Table 3:** Shoot characteristics and time of harvest for individuals studied (means ± se).

Species	Family	n	Height of plant crown (cm)	Width of plant crown (cm)	Width of collar diameter Dc (cm)	Mode of reproduction	Months when roots were collected (total precipitations for the month)
*Agava americana*	Asparagaceae	14	16.81 ± 20.45	22.91 ± 39.77	0.43 ± 0.56	vegetative	July 2010 (275 mm)
*Artemisia codonocephala*	Asteraceae	12	12.74 ± 4.37	22.27 ± 8.45	0.75 ± 0.35	sexual	May 2009 (97 mm) and July 2010 (275 mm)
*Arthraxon hispidus*	Poaceae	12	17.25 ± 11.29	15.00 ± 6.15	0.43 ± 0.16	sexual, vegetative	June (181 mm) and July 2010 (275 mm)
*Bauhinia championii*	Leguminosae	12	20.82 ± 12.38	27.77 ± 28.20	0.61 ± 0.30	sexual, vegetative	June (181 mm) and July 2010 (275 mm)
*Chloris anomala*	Poaceae	12	11.78 ± 6.92	17.18 ± 6.18	5.41 ± 6.70 (tufts)	sexual, vegetative	May 2009 (97 mm) and June 2010 (181 mm)
*Ficus tikoua*	Moraceae	11	7.60 ± 3.03	36.38 ± 31.27	0.71 ± 0.33	sexual	August 2009 (137 mm) and July 2010 (275 mm)
*Jatropha curcas*	Euphorbiaceae	5	22.80 ± 9.39	15.30 ± 10.77	0.17 ± 0.06	sexual	July 2010 (275 mm)
*Pueraria stricta*	Leguminosae	12	33.75 ± 26.06	22.98 ± 11.32	0.41 ± 0.17	sexual	May 2009 (97 mm) and June 2010 (181 mm)
*Rhus chinensis*	Anacardiaceae	13	16.54 ± 8.73	22.54 ± 11.46	0.50 ± 0.23	sexual, vegetative	August 2009 (137 mm), June (181 mm) and July 2010 (275 mm)

During excavation, the orientation of roots in a circular sector was considered qualitatively: all roots in the upper half of the sector, with regard to slope direction, were noted as upslope roots, and those in the lower sector noted as downslope roots. Roots were also classed into depth classes of 10 cm perpendicular to the soil surface. During excavation, roots were covered with wet towels to prevent desiccation. Root systems were then transported to the laboratory and stored at 4°C.

### Architectural traits

Soil volume was calculated as the corresponding portion of individual soil volume (ISV) in each 10 cm layer of soil and in each upslope or downslope sector. Spatial x, y, z coordinates of each structural root allowed us to determine the maximum radius and depth of each root system. Using these coordinates for each plant, ISV was calculated as a quarter of ellipse upslope plus a quarter of ellipse downslope, with the size of the ellipse determined by the maximum radius and depth. However, in *A. americana* and *F. tikoua*, root systems were organised linearly along the main plant stem (Figure 3). Therefore, the ISV of these species was calculated as a quarter of a cylinder upslope plus a quarter of a cylinder downslope. The maximum radius and ISV volume were then deduced for each soil layer from geometrical equations. Standardizing ISV by the collar diameter allows plant individuals of different sizes to be compared [55].

Root area ratio (RAR) is the cumulated cross sectional area (CSA) of all roots crossing the potential shear surface per unit of soil surface. RAR can also be calculated as the cumulated volume of roots per unit of soil volume, if we consider that all roots within a layer cross the surface of this layer perpendicularly [56].
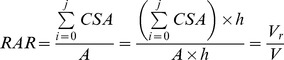
(Eq. 4)where 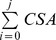: sum of CSA in diameter classes [0; j] of all roots crossing a soil surface *A*, *h*: height of the considered layer, *V_r_*: cumulated volume of roots within the corresponding volume of soil *V*.

RAR was calculated for roots separated into two diameter classes: fine [0; 2 mm], and coarse [2 mm; + 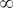]. Within 24 hours after harvesting, roots were washed, paper-dried and scanned at a resolution of 700 dpi using an EPSON V700 Pro scanner. Root length, diameter and volume were measured using the image analysis software WinRHIZO (Pro version 3.0, Regent Instruments, Canada) [57], within each 10 cm depth class and within up- and down-slope sectors.

### Mechanical traits

After scanning, and within 24 hours after harvesting, tensile testing of a sub-sample of individual roots was performed for each species. Testing was successfully carried out on 1116 roots, using a portable testing machine (In-Spec 2200 BT, Instron Corporation) equipped with three force transducers (maximum capacities of 250, 50 and 10 N and accuracy of 0.25%) chosen according to the size of the root. Span tests were carried out for each species to check whether the length of each sample had to be at least 30 times its central diameter [58]. Root diameter was measured at three points along each root using a binocular microscope and the mean diameter calculated. Crosshead speed was kept constant at 1.0 mm min^−1^ and both force and speed were measured constantly during each test (Instron Series IX software). Tests to failure were considered successful only when specimens failed in the middle third of the root and if the root did not slip inside the clamps, because we were measuring tensile stresses as well as tensile strains. In order to avoid slippage of roots out of the clamps, the clamps were chosen according to the diameter of the root and two pieces of sandpaper were placed on either side of the root within the jaws of the clamp. Each stress-strain curve was analysed to obtain the mechanical properties of the root [12], [21], in particular its maximal tensile stress (T_max_) and the ultimate strain at failure (ε_ult_). T_max_ was calculated as the force required to cause breakage, divided by the root CSA at the point of breakage. ε_ult_ was calculated as the displacement between the clamps at breakage divided by the initial distance between the clamps. For measuring bending properties of thick roots, three-point bending tests were performed. Span tests were carried out for each species to check the necessary span to depth ratio in order to avoid including significant amounts of shear [59]. The depth diameter (*d*) and the width diameter (*w*) were measured at three points along the root using a binocular microscope. The axial second moment of inertia (*I*) was calculated using:
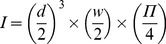
(Eq. 5)


The stiffness or bending modulus of elasticity *E* was calculated from the stress-strain curve of 137 bending tests as the linear slope shortly after the beginning of the test [60]. The bending modulus is not equal to the above-cited tensile modulus of elasticity [61]. The bending rigidity (*EI*) of each sample was calculated as the product of 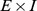.

### Physiological traits

After mechanical testing, roots were dried at 40 °C until constant weight. Nitrogen concentration on seven (*A. hispidus*) up to 77 (*B. championii*) roots per species was determined using an elemental analyser (CHN model EA 1108; Carlo Erba Instruments, Milan, Italy). The concentration of cellulose was measured on six (*A. hispidus*) up to 50 (*B. championii*) roots per species, using the Van Soest method [62], and with a Fibersac 24 fiber analyser (Ankom, Macedon, NJ, USA). The number of analysed roots per species depended on the quantity of available material for each species: the amount of collected roots was very light for some species (e.g. *A. hispidus*) whereas they were much heavier for other species (e.g. *B. championii*).

### Statistical analysis

Different statistical tests were used depending on the number and type of factors (Table 4). In the majority of analyses, root diameter was considered a continuous variable. In some analyses, root diameters were separated into two classes: fine [0; 2 mm], and coarse [2 mm; + 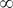] and are indicated as “diameter class”.

**Table 4:** Different statistical tests were used depending on the variables tested.

Statistical test	Presentation of results and parameters	Analyzed data
Linear correlation between two continuous variables	r = correlation coefficient, R^2^ = r^2^ = coeff of determination	Log(T_max_) and ε_ult_ depending on diameter
Anova: parametric test of analysis of variance	F _factor df, errors df_ value; P value	N and CELL depending on species and diameter classes
Ancova: parametric test of analysis of covariance with root diameter as covariable	Fcov _factor df, errors df_ value; P value	T_max_, ε_ult_ and *EI* depending on species; T_max_ and ε_ult_ depending on depth, up/downslope, sites and season
Kruskal-Wallis non parametric test for independant variables	H _n = nb cases, N = nb of observations_ value; P value	Number of stems m^−2^, ISV/Dc and RAR depending on species; *EI*, N and CELL depending on depth
Friedman Anova non parametric test for dependant variables (>2)	X^2^_N = nb of observations, factor df_ value; P value	RAR depending on depth
Mann-Whitney U non parametric test for two independant variables	Z _nb of valid observations in one case, nb of valid observations in the other case_ value; P value	*In situ* soil resistance, *in situ* soil penetration, Number of stems m^−2^, ISV/Dc, RAR, EI, N and CELL depending on sites; Number of stems m^−2^, ISV/Dc and RAR depending on seasons

Abbreviations and parameters used in the text are also indicated.

Each time parametric tests are used, the following assumptions were checked: residuals are independent, they have homogeneous variance (homoscedasticity) and they are identically distributed following the normal law N (0, σ^2^). If these assumptions were not met, parametric tests were still performed on raw data if the amount of data was >30 in each treatment within each data set (Central Limit Theorem) [63]. Data were not log-transformed because of the implied data distortion [63]. Post-hoc tests used Tukey Honestly Significant Difference (HSD) tests to discriminate among treatments. When parametrical tests could not be used, non-parametrical tests were performed (Table 4).

### Scores for species

We aimed at designating scores for each species with regard to their utility as ‘ecological engineers.’ The traits of each species were assigned a score depending on their suitability for fixing soil on slopes: for each trait, a score equal to 1 (poor performance), 2 (average), 3 (good performance) was attributed to each species. A global score was then attributed for each of the three studied properties: (i) ability to occupy soil with roots; (ii) mechanical resistance; and (iii) root physiological properties (Table 1). To obtain a more accurate estimation for root resistance in tension, the score obtained for the maximal tensile strength was multiplied with the score obtained for the proportion of fine roots (RAR_f_). Similarly, to determine a more accurate estimation for root resistance in bending, the score obtained for *EI* was multiplied with the score obtained for the proportion of coarse roots (RAR_c_).

The rules used for synthesizing scores were:

Poor performance (score 1) combined with poor performance (1)  =  poor global performance (1);

Good performance (3) combined with good performance (3)  =  good global performance (3);

Poor performance (1) combined with good performance (3)  =  average global performance (2);

Poor performance (1) combined with average performance (2)  =  poor global performance (1);

Good performance (3) combined with average performance (2)  =  good global performance (3).

## Results

### Soil characteristics

In situ shear resistance (measured using a tore vane) was significantly lower in the hotspot compared to the stable site (Z_20,20_ = −3.28, P<0.001; Table 2). In situ resistance to penetration was significantly lower at the hotspot (Z_20,20_ = −5.25, P<0.001; Table 2). Soil cohesion (measured via direct shear tests in the laboratory) was extremely low at both sites, in particular the hotspot (Table 2), whereas the angle of internal friction was similar between sites (Table 2).

### Species abundance

**Figure 4:**
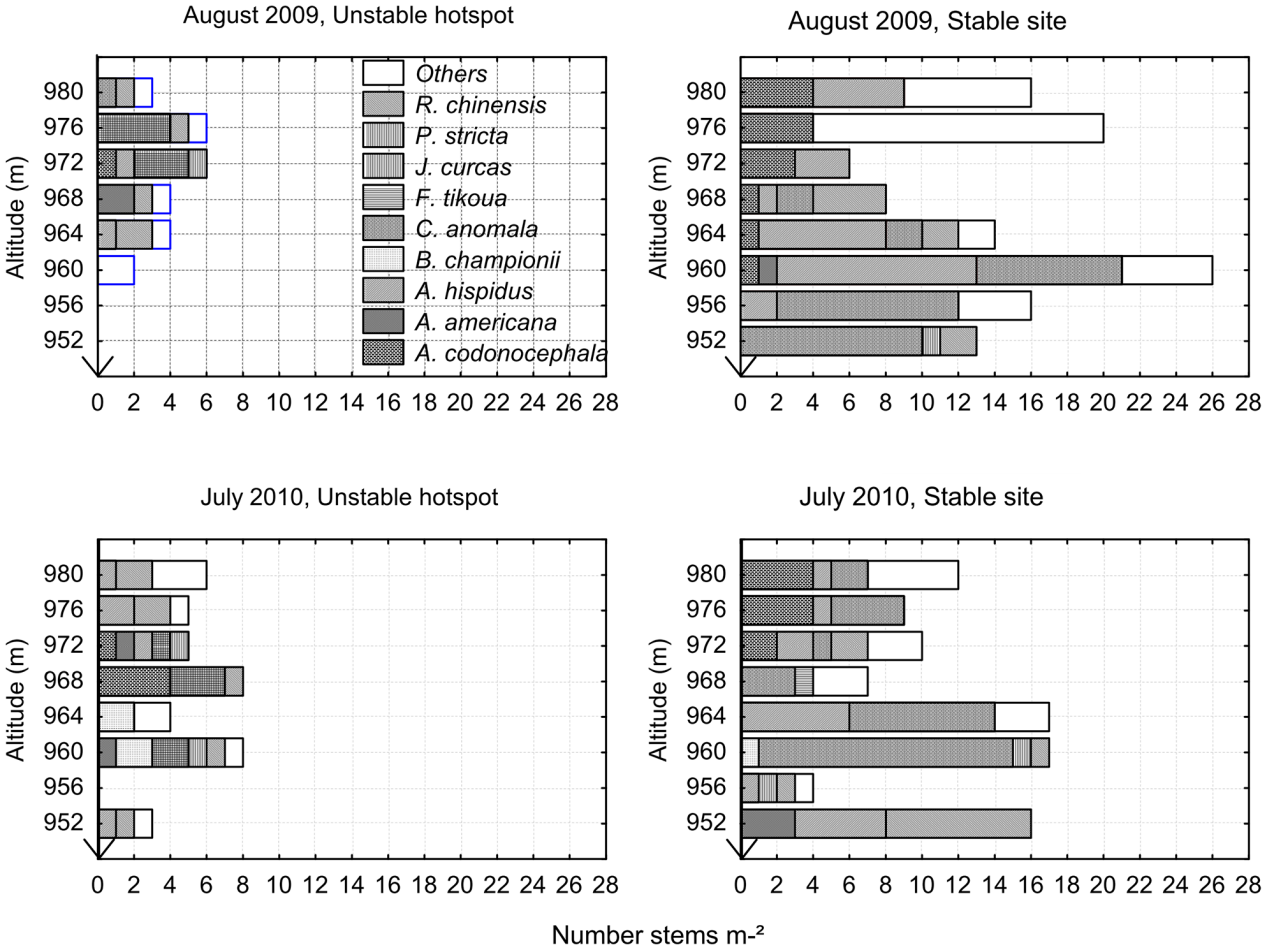
Plant abundance. Number of stems per square meter of each species present on the unstable hotspot and the stable site in 2009 and 2010. “Others” were composed of the following species: *Bidens pilosa* L., *Celosia argentea* L., *Elsholtzia winitiana* Craib, *Indigofera* sp., *Malvastrum coromandelianum* (L.) Garcke, *Convolvulus arvensis* L., *Solanum verbascifolium* L.

Regardless of site and year, the most abundant species were the two herbaceous species *C. anomala* and *A. hispidus*, followed by the creeping liana *F. tikoua* and then the tree *R. chinensis* (H_9,460_ = 95.88; P<0.001; Figure 4). All four species were observed to reproduce through clonal reproduction. *A. codonocephala* was relatively abundant. *A. americana*, *J. curcas* and *P. stricta* had been planted on the same slope where our fieldsites were located but at a lower altitude. Nevertheless, results showed that these species had begun to spread up the slope and colonize our sites. *P. stricta* spreads through sexual reproduction, producing numerous, light seeds [64], whereas *A. americana* reproduces largely through the production of underground stems [65]. *J. curcas* reproduces through large and heavy seeds when adult [66]. *B. championii* was not highly abundant (Figure 4).

*A. codonocephala, A. hispidus* and *C. anomala* produced a significantly higher number of stems m^−2^ at the stable site compared to the unstable hotspot (H_2,46_ = 6.36; P = 0.042; H_2,46_ = 7.49; P = 0.024 and H_2,46_ = 12.13; P = 0.002 respectively). On the contrary, *F. tikoua* possessed a significantly higher number of stems m^−2^ at the unstable hotspot compared to the stable site (H_2,46_ = 6.54; P = 0.038). *B. championii* produced a higher number of stems m^−2^ in July 2010 (very wet period) compared to August 2009 (dryer period; Z_22,24_ = −2.46; P = 0.013). No other differences were observed with regard to site or year.

### Root spread (ISV/Dc)

**Figure 5:**
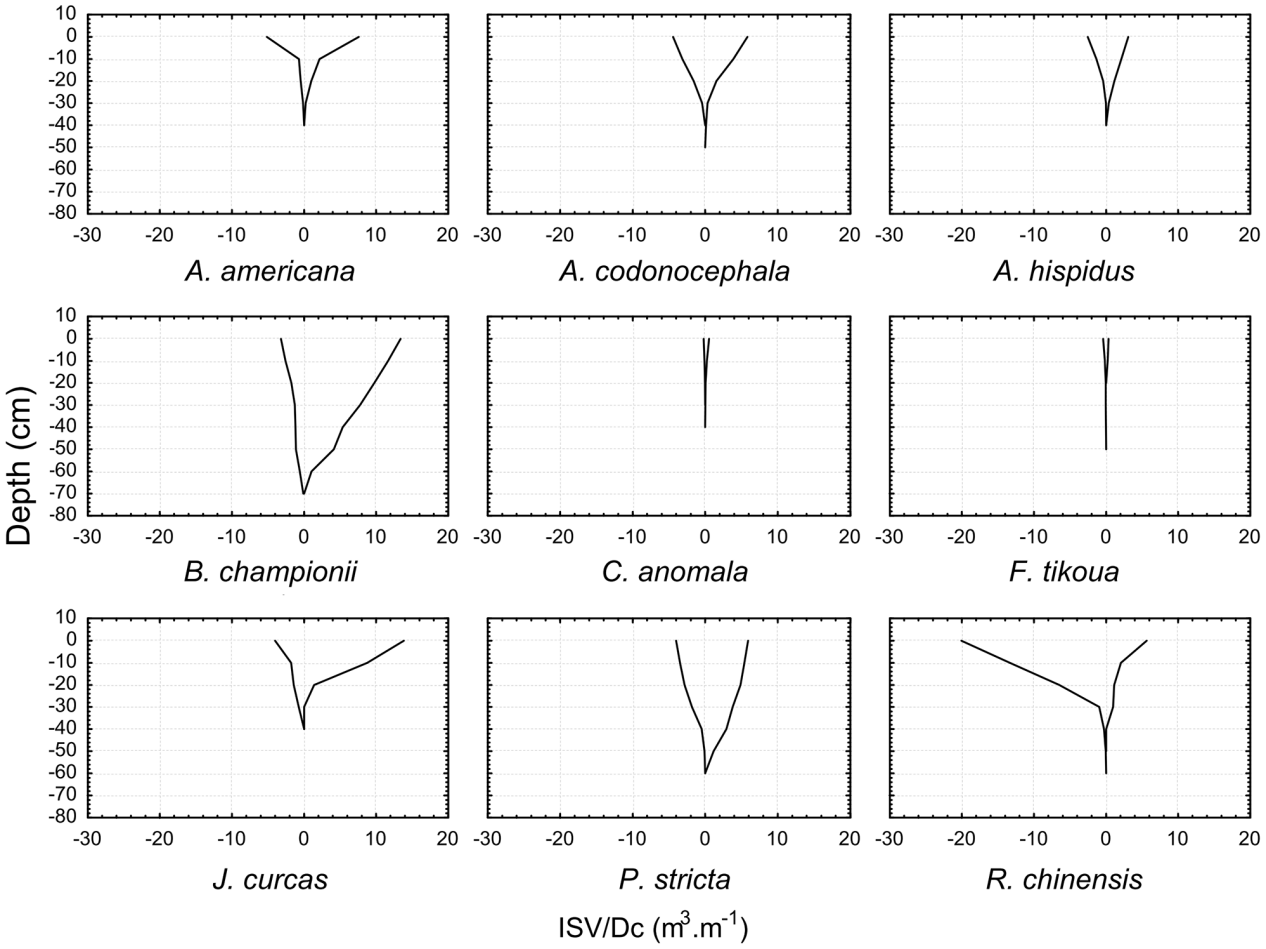
Mean individual soil volume standardized by collar diameter (ISV/Dc). Negative values of ISV/Dc represent downslope orientation.

When standardized by collar diameter (Dc), ISV depended on species (H_8,104_ = 54.44; P<0.001; Figure 5). In particular, *B. championii* possessed deeper and wider ISV/Dc compared to other species. *P. stricta*'s root system was also very deep (up to 60 cm). *F. tikoua* and *C. anomala* root systems occupied a very narrow ISV/Dc (Figure 5).

*B. championii, C. anomala* and *P. stricta* occupied a significantly higher ISV/Dc upslope compared to downslope from the stem (Z_13_ = 2.20; P = 0.028; Z_12_ = 3.06; P = 0.002 and Z_12_ = 1.80; P = 0.007 respectively, Figure 5). *R. chinensis* exploited a significantly larger ISV/Dc at the unstable hotspot compared to the stable site (Z_6,7_ = −1.93; P = 0.005, Figure 5). No other differences were observed with regard to site or slope sector.

### Root area ratio (RAR)

**Figure 6:**
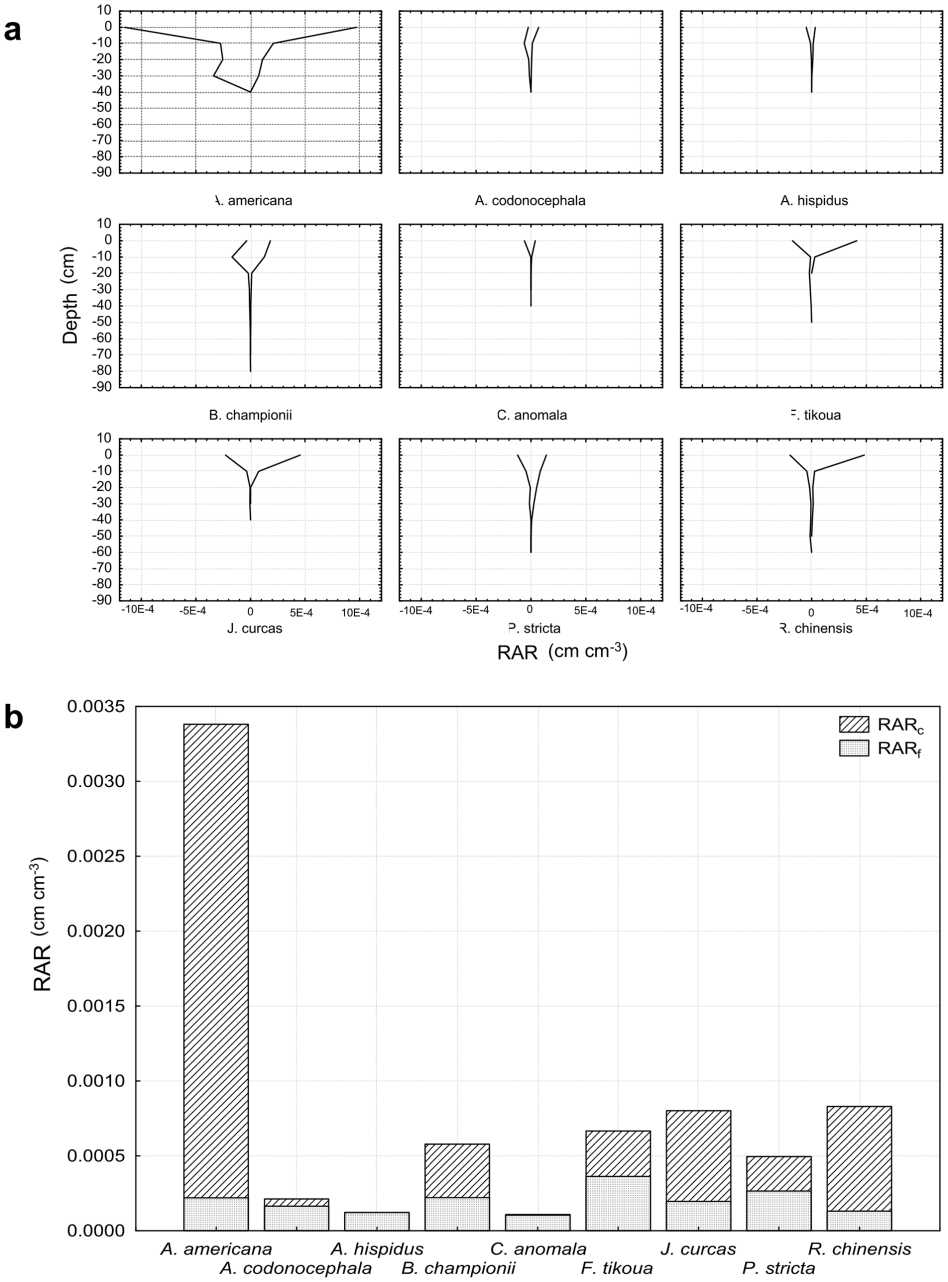
Root density of all species and proportion of coarse and fine roots. (a) Root area ratio (RAR) of all species. Negative values of RAR represent downslope orientation; (b) Coarse and fine roots area ratio (RAR).

RAR was species-dependant (H_8,100_ = 50.25; P<0.001; Figure 6a), with *A. americana* having significantly higher RAR than all other species. When *A. americana* was removed from the analysis, the species effect was still significant (H_7,86_ = 38.78; P<0.001): *F. tikoua, J. curcas, B. championii, P. stricta* and *R. chinensis* all possessed significantly higher RAR than *A. codonocephala*, *A. hispidus* and *C. anomala* (Figure 6a). *B. championii, P. stricta* and *R. chinensis* all produced roots at depth of >50 cm.

For all species, RAR decreased significantly with increasing depth (Χ^2^_14,3_ = 14.36; P = 0.002 for 0–10 cm compared to 10–20 cm and X^2^_12,7_ = 68.97; P<0.001 for 10–20 cm compared to 20–30 cm, Figure 6a) except for *A. americana* and *B. championii,* for which RAR at 10–20 cm was not significantly lower than RAR at 0–10 cm. *P. stricta* possessed a significantly higher RAR in the upslope compared to downslope sector (Z_12_ = 1.65; P = 0.009, Figure 6a). *A. codonocephala* had a higher RAR at the unstable hotspot compared to the stable site (Z_4,6_ = 2.02; P = 0.043). No other significant differences between depth, slope sector or site were found.

With regard to the RAR of fine and coarse roots, the RAR of coarse roots was significantly greater in *A. americana* (Figure 6b) because we made no distinction between coarse roots and underground stems. The proportion of coarse roots was also higher than the proportion of fine roots for *J. curcas* and *R. chinensis*. *A. codonocephala, A. hispidus* and *C. anomala* root systems were composed largely of fine roots, therefore RAR of coarse roots was low (Figure 6b). For *B. championii*, *F. tikoua* and *P. stricta*, RAR of fine and coarse roots were similar (Figure 6b).

### Root strength in tension (T_max_)

**Figure 7:**
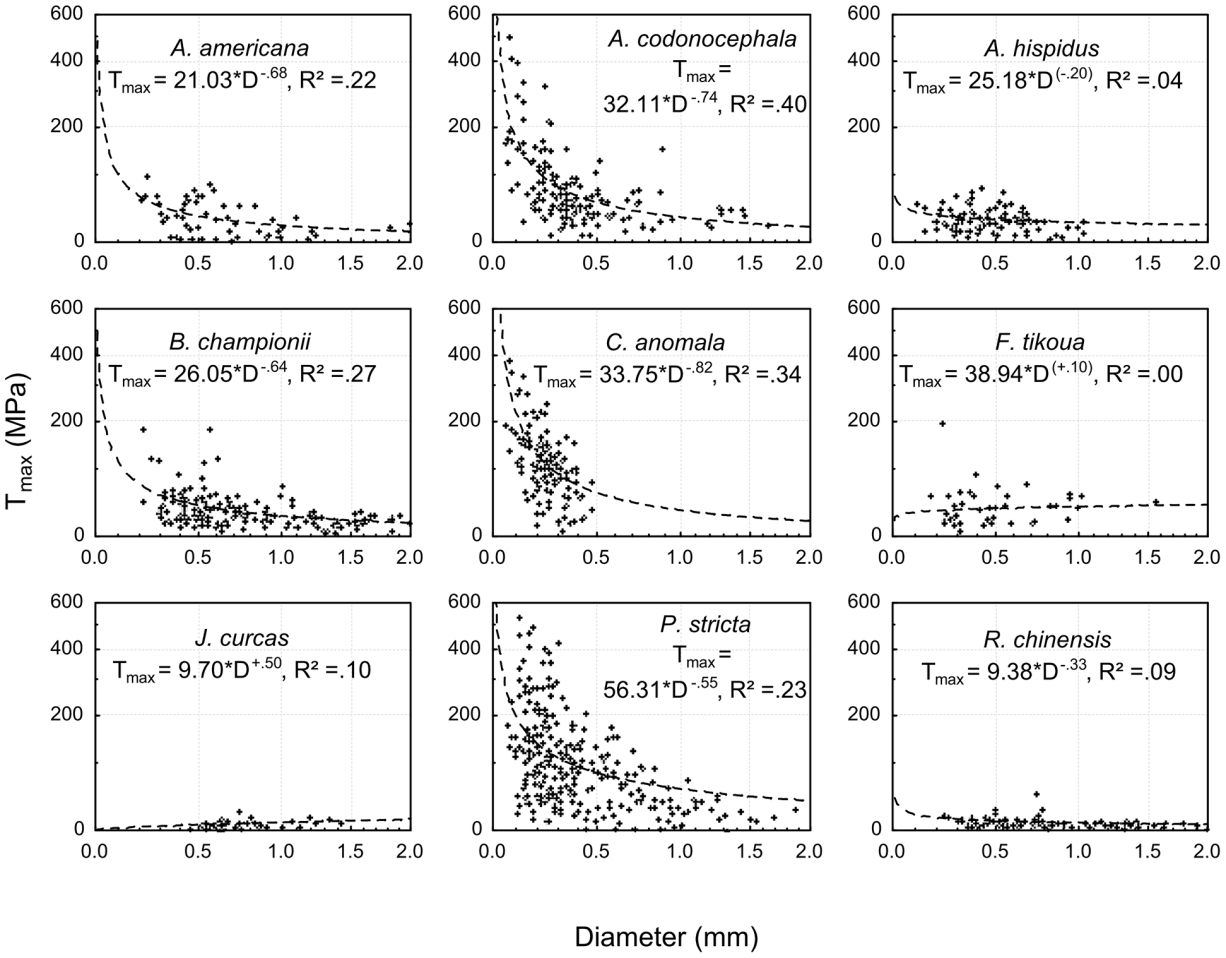
Tensile stress at failure. Tensile stress at failure (T_max_) for root diameters from 0 to 2 mm. Logarithmic scales. Fitting curves: T_max_  =  α*D^−β^, equations are presented on the graphs with the determination coefficient (R^2^), parameters in brackets are not significant.

T_max_ increased with decreasing root diameter in all species except *F. tikoua* and *J. curcas* in which T_max_ increased with increasing root diameter (Figure 7). These trends were not significant for *A. hispidus* and *F. tikoua*. T_max_ differed significantly between species when root diameter was used as a covariate (Fcov_9,1105_ = 29.85; P<0.001; Figure 7). All species possessed similar values for the T_max_ of coarse roots (15–20 MPa) but T_max_ was greater in coarse roots of *F. tikoua* and *P. stricta* (approximately 40 MPa) and lower in coarse roots of *J. curcas* and *R. chinensis* (7–15 MPa; Figure 7). For fine roots, *P. stricta* had the strongest T_max_ (up to 200 MPa for roots 0.1 mm in diameter), followed by *A. codonocephala* and *C. anomala*. *J. curcas* and *R. chinensis* possessed very low T_max_ for fine roots (Figure 7).

With regard to depth in the soil, roots <1 mm in diameter of *B. championii* growing at a depth >30 cm possessed a significantly greater mean T_max_ compared to roots growing in shallower depth layers (Fcov_1,192_ = 3.91; P<0.001). For a given diameter, roots of *A. americana* and *C. anomala* were significantly stronger at the stable site compared to the unstable hotspot (Fcov_1,50_ = 14.66; P<0.001 and Fcov_1,118_ = 4.18; P = 0.043 respectively). For *P. stricta*, roots thicker than 0.5 mm diameter had a higher mean T_max_ at the unstable hotspot compared to the stable site but the tendency was inversed for roots thinner than 0.5 mm (Fcov_1,286_ = 5.21; P = 0.023). *R. chinensis* possessed higher mean T_max_ for roots >1.5 mm diameter at the unstable hotspot than at the stable site and again the tendency was inversed for thinner roots (Fcov_1,93_ = 6.80; P = 0.011). With regard to the season when the roots were harvested for mechanical testing, roots of *A. codonocephala*, *C. anomala* and *P. stricta* were significantly stronger during the dry season (May 2009) compared to the rainy season (July 2010; F_1,46_ = 26.61; P<0.001; Fcov_1,118_ = 14.74; P<0.001 and Fcov_1,286_ = 12.31; P<0.001 respectively). No other differences in mean T_max_ were found with regard to depth, slope sector, field sites and the season when roots were harvested.

### Root strain in tension (ε_ult_)

**Figure 8:**
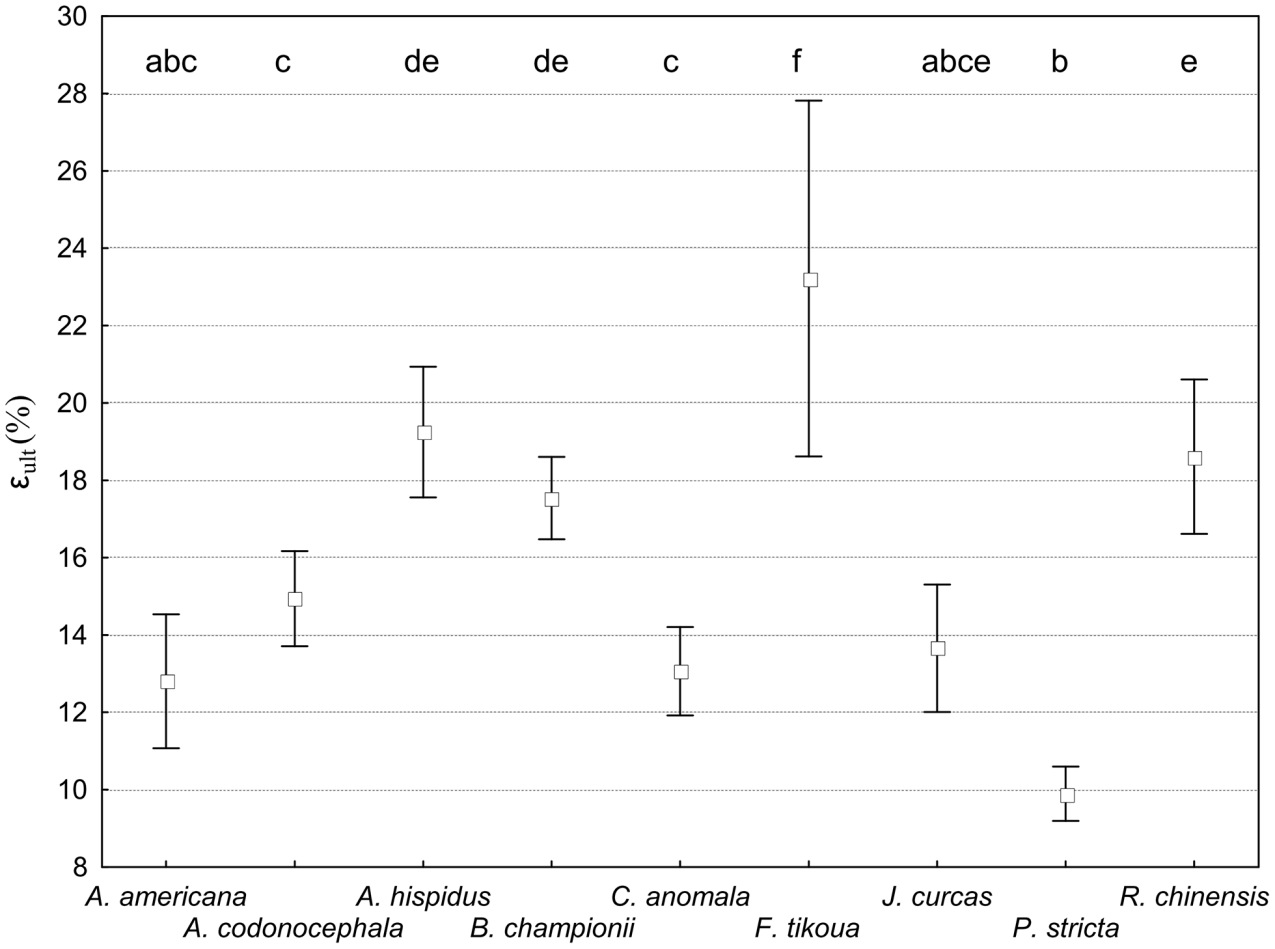
Ultimate strain at failure. Ultimate strain at failure (ε_ult_) for each species. Vertical bars denote 0.95 confidence intervals, letters indicate significant differences between species (P<0.05).

The mechanical behaviour of roots differed between species with regard to strain. A positive relationship between ε_ult_ and root diameter was significant for *A. codonocephala* (r = 0.23, P<0.001) and *P. stricta* only (r = 0.48, P<0.001). For all other species, correlations between ε_ult_ and root diameter were not significant. Mean ε_ult_ was significantly different depending on species (F_8,1105_ = 32.34; P<0.001; Figure 8). *F. tikoua* had the highest ε_ult_ (mean ε_ult_ = 23,24%), followed by *A. hispidus*, *B. championii* and *R. chinensis* (Figure 8). *A. americana*, *A. codonocephala*, *C. anomala, J. curcas* had relatively small mean ε_ult_ with *P. stricta* having the the smallest ε_ult_ (mean ε_ult_ = 9,81%).

Roots of *A. codonocephala* had significantly smaller mean ε_ult_ at depths >30 cm compared to roots growing at depths <30 cm (Fcov_4,146_ = 3.65; P<0.001). On the contrary, in both *P. stricta* and *R. chinensis*, mean ε_ult_ was significantly greater in roots located at depths >30 cm than in shallow roots (Fcov_5,286_ = 5.64; P<0.001 and F_5,91_ = 3.93; P = 0.003, respectively). With regard to differences between upslope and downslope sectors, mean ε_ult_ in roots of *P. stricta* was significantly shorter upslope compared to downslope (Fcov_1,286_ = 5.88; P = 0.016). Roots of *P. stricta* possessed a mean ε_ult_ that was significantly smaller at the unstable hotspot compared to the stable site (Fcov_1,286_ = 6.76; P = 0.023) and also significantly smaller during the dry season (May 2009) compared to the rainy season (July 2010; Fcov_1,286_ = 9.75; P = 0.002). On the contrary, roots of *A. hispidus* possessed significantly smaller mean ε_ult_ in July 2010, compared to the drier month of June 2010; F_1,89_ = 295.29; P = 0.041). No other significant differences in mean ε_ult_ were found with regard to depth, slope sector, site and the season when roots were harvested.

### Resistance in bending (*EI*)

**Figure 9:**
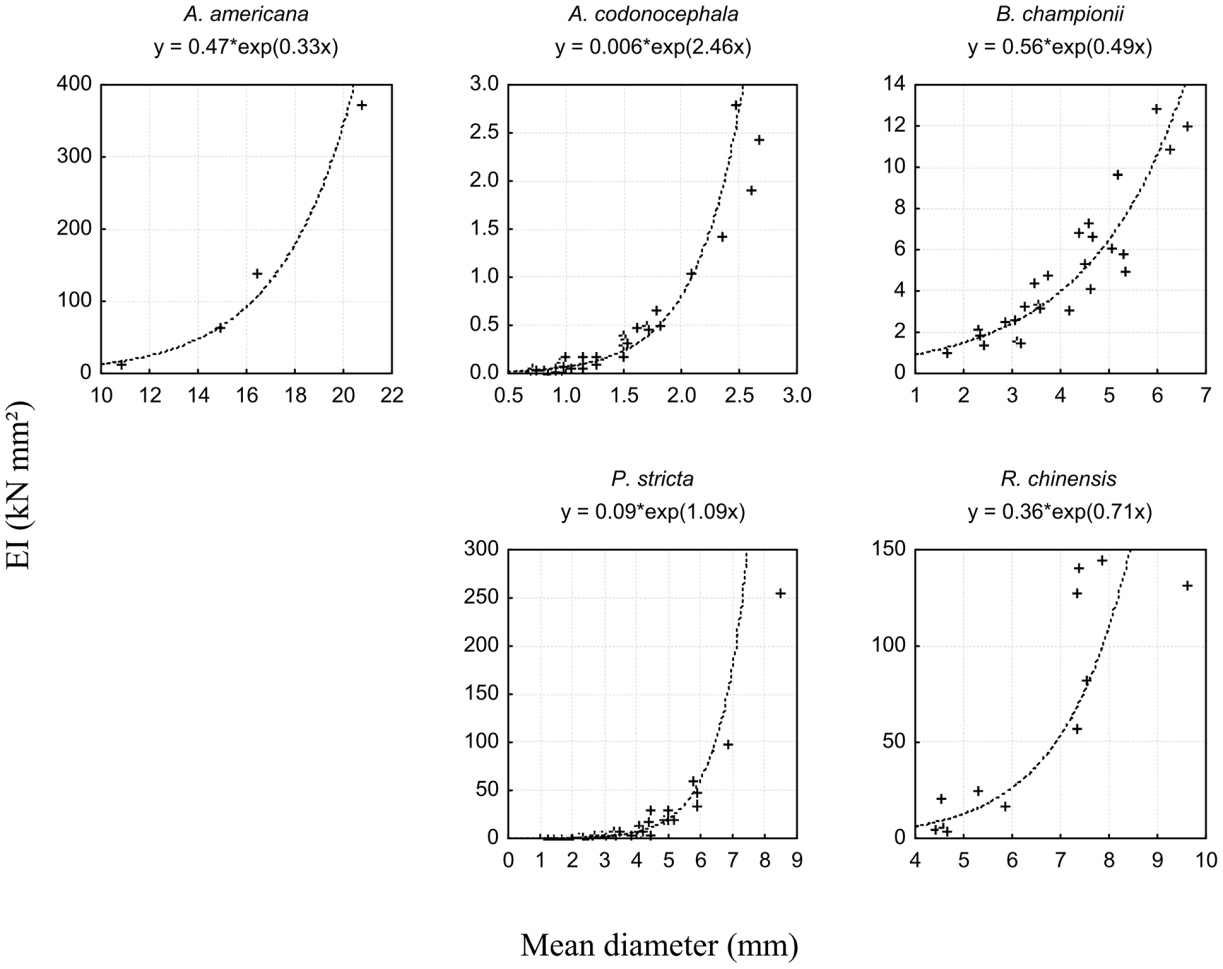
Bending rigidity. Bending rigidity (*EI*) as a function of root mean diameter for each species where data were sufficient for statistical analyses. Mean root diameter is the mean of depth and width diameters for each root. Note that scales differ between graphs for easier viewing.

In species where enough large and stiff roots were found, i.e. all species except *A. americana*, *A. hispidus*, *C. anomala, F. tikoua* and *J. curcas*, *EI* increased with root diameter (Figure 9). *EI* differed significantly between species when root diameter was used as a covariate (Fcov_6, 128_ = 240.41; P<0.001; Figure 9). Roots which were the most resistant in bending belonged to *P. stricta* (300 kN mm^2^), *R. chinensis* (maximal values around 1.50 kN mm^2^), followed by *B. championii* (15 kN mm^2^) and *A. codonocephala* (3 kN mm^2^).

The only significant difference with regard to site was found for roots of *P. stricta*, where mean *EI* was higher at the unstable hotspot compared to the stable site (Z_21,5_ = −2.08; P = 0.037). Data were too few to obtain significant comparisons between root depth in the soil and slope sector.

### Chemical composition

**Figure 10:**
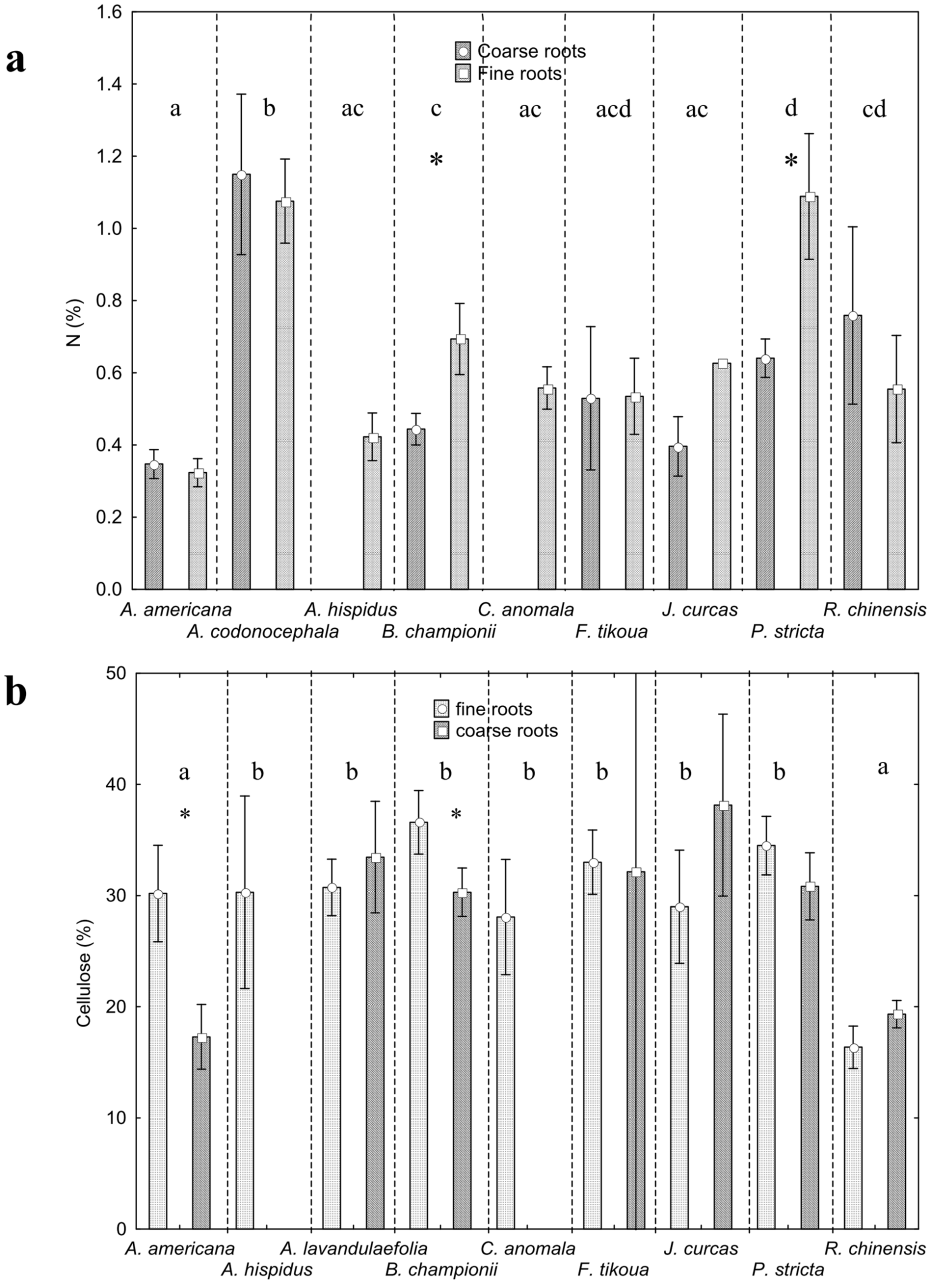
Nitrogen and cellulose contents. a) Nitrogen and b) cellulose concentrations in coarse and fine roots for each species. Letters indicate significant differences between species (P<0.05) when all (coarse and fine) roots are taken into account. Asterisks denote significant differences between coarse and fine roots within a species. Vertical bars denote 0.95 confidence interval.

The quantity of N present in roots depended on species (F_8,231_ = 33.48; P<0.001, Figure 10a) and the interaction between species and root diameter (F_15,224_ = 26.32; P<0.001, Fig; 10a). *A. codonocephala* had the highest quantity of N present in roots, regardless of root diameter (Figure 10a). The leguminous *P. stricta* also possessed high levels of N in fine roots (Figure 10a), as did the fine roots of the leguminous *B. championii*, but to a lesser exent (Figure 10a).

With regard to slope sector, all roots of *A. codonocephala* growing downslope contained more N than those growing upslope (Z_9,7_ = −2.12; P = 0.034). In *J. curcas*, N was significantly higher in roots growing upslope compared to those growing downslope (Z_5,5_ = 2.30; P = 0.021). With regard to site, root N was significantly greater in all roots of *A. americana*, *B. championii*, *P. stricta* and *R. chinensis* growing on the unstable hotspot (Z_17,32_ = 4.19; P<0.001; Z_24, 44_ = 3.29; P<0.001 and Z_29,21_ = 3.54; P<0.001; Z_5,6_ = 2.46; P = 0.014 respectively). No other differences in root N were found with regard to slope sector or site and no differences were found with regard to depth.

The quantity of cellulose in roots depended on species (F_8,214_ = 20.90; P<0.001, Figure 10b) and on the interaction between species and root size (F_6,333_ = 8.33; P<0.001, *A. hispidus* and *C. anomala* having no coarse roots and root size not being defined for some samples, Figure 10b). For all species, cellulose content was significantly higher in fine roots compared to coarse roots (F_169.02_ = −4.55; P<0.001), *A. americana* and *B. championii* having significantly higher cellulose in fine roots than in coarse roots (F_8,214_ = 20.903; P<0.01 and F_6,190_ = 8.33; P<0.01 respectively). The highest cellulose content was found in coarse roots of *J. curcas.* The lowest cellulose content was found in fine and coarse roots of *R. chinensis* and in coarse roots (i.e. coarse roots and underground stems) of *A. americana* (Figure 10b). With regard to root depth in the soil, *P. stricta* possessed significantly more cellulose in roots of at a depth 0.0 to 0.10 m compared to those deeper than 0.10 m (H_3,42_ = 0.00; P = 0.014). The only differences between sites were in roots of *A. americana,* which had more cellulose at the stable site compared to the unstable hotspot (Z_28,13_ = 3.07; P = 0.002). No other significant differences in cellulose content were found with regard to depth, site and upslope/downslope sectors.

## Discussion

### Differences in traits between species and sites

**Figure 11:**
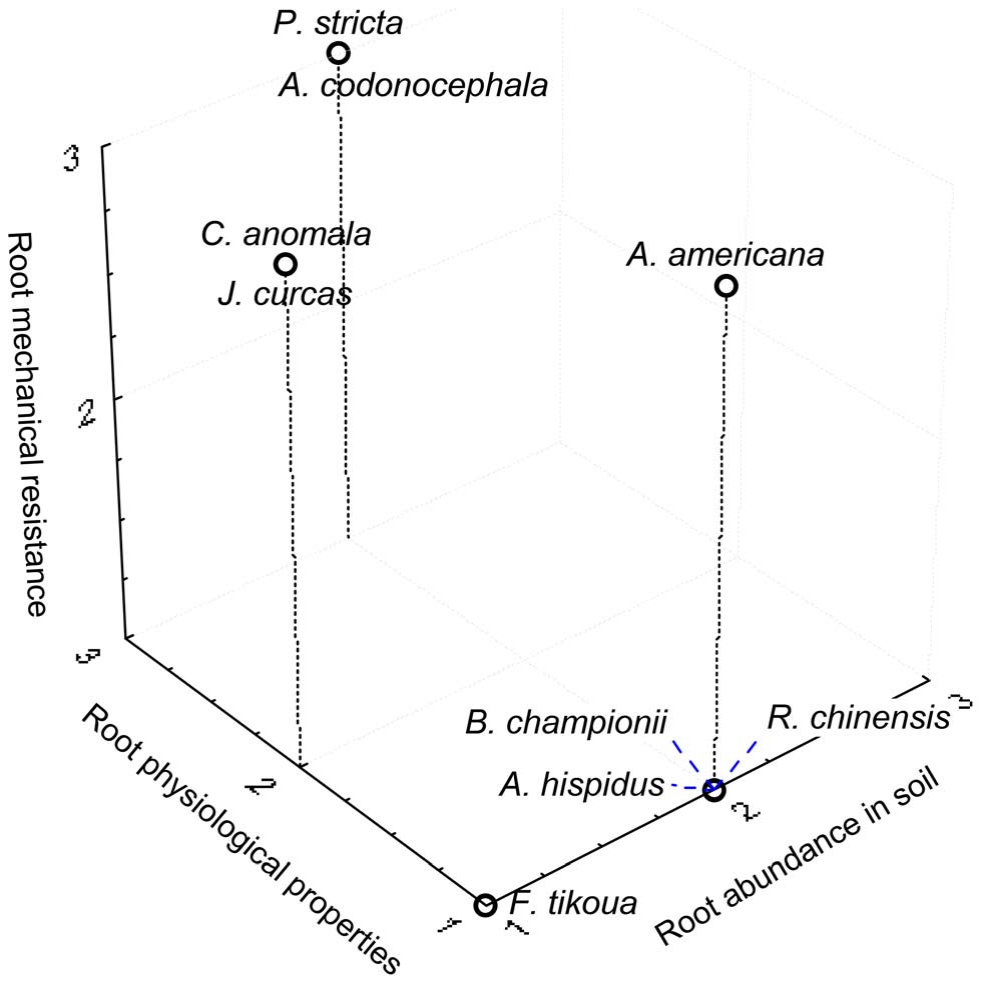
3D diagram of species' scores. Summary of species' performance according to root abundance in soil, root mechanical resistance and root physiological properties.

The traits of each species were examined with regard to their desirability for fixing soil on slopes (Table 5 and Figure 11). Each species possessed one or several traits, which were desirable for improving slope stability, but no one species possessed a suite of traits that were ideal for fixing soil. *P. stricta* and *A. codonocephala* obtained the highest scores, as they possessed roots which were mechanically resistant and had suitable physiological traits, but soil occupation was average (Table 5 and Figure 11). In the case of *P. stricta*, the poor occupation of soil by roots was mainly due to a low number of stems per square meter. It can be assumed that increasing the number of individuals would increase root density in the soil over time. *J. curcas* and *A. americana* have been planted at many sites in the world to counteract slope instability and erosion processes [67], [68], [69]. However we found that although root mechanical properties were suitable, these species were not among the most useful for reinforcing soil (Table 5 and Figure 11), because of a poor capacity to occupy soil (*J. curcas*) and unsuitable physiological traits (*A. americana*). *A. hispidus* (herb), *B. championii* (leguminous liana) and *R. chinensis* (tree with vegetative multiplication by roots) had poor scores. They did not possess the same root system morphologies (Figure 3). All three species had poor mechanical and physiological root traits, and only an average soil occupation. The scoring system we developed allows us therefore to consider very different species and estimate their performance with regard to slope stability.

**Table 5:** Multi-criteria table summarizing performances of each species: traits and related functions.

Functional traits & related functions	*A. americana*	*A. codonocephala*	*A. hispidus*	*B. championii*	*C. anomala*		*F. tikoua*			*J. curcas*			*P. stricta*		*R. chinensis*
High presence on hotspots (high number stems m^-2^)	1	2	3	1	3		2			1			1		2
High propagation of roots (high ISV/Dc)	2	2	2	3	1		1			2			3		2
High root density (high RAR)	3	2	1	2	1		2			2			2		2
**Root abundance in soil**	2	2	2	2	1	1			1			2			2
High resistance in tension (high fine roots RAR x high T_max_)	1*2 = 1	3*3 = 3	3*2 = 3	2*2 = 2	3*3 = 3	2*2 = 2			1*1 = 1			2*3 = 3			1*1 = 1
High resistance in bending (high coarse roots RAR x high *EI*)	3*3 = 3	1*2 = 1	1*1 = 1	2*2 = 2	1*1 = 1	2*1 = 1			3*3 = 3			2*3 = 3			3*3 = 3
Short deformation (short ε_ult_)	3	3	1	1	3	1			3			3			1
**Root mechanical resistance**	3	3	1	1	3	1			3			3			1
Short deformation (short ε_ult_)	2	2	2	2	2	1			2			3			2
High metabolism (high N)	1	3	1	2	1	1			1			3			2
High longevity (high CELL)	2	3	1	1	3	3			3			1			1
**Root physiological properties**	1	3	1	1	2	1			2			3			1

1/2/3: poor/average/good performance.

### Seasonality and root traits

Several species possessed different properties depending on the season. Among them, *B. championii* did show an increase in stem abundance from August 2009 to July 2010, but this was probably due to progressive colonization of the slope rather than a seasonal effect. The mechanical resistance of roots of *A. codonocephala*, *C. anomala* and *P. stricta* (estimated by an increase of T_max_ in the three species and a decrease of ε_ult_ in *P. stricta*) decreased during the wet season. However, [24] showed that during winter and wet conditions, the tensile strength of *Salix* and *Populus* clones was higher. The only species with a slight advantage during the monsoon season was *A. hispidus*, whose roots deformed less in the wetter conditions. Mao et al., [70] showed that fine roots of temperate tree species produced during the summer were thinner and more short-lived than those produced during the winter months. The investment of resources in short-lived roots is usually less than that in longer-lived roots [32]. Therefore, the differences in mechanical properties that we observed are probably due to inherent differences between species and linked to seasonality in root production. The chemical composition of roots will also influence mechanical properties depending on water content.

### Which species for slope short-term restoration?

All species showed significant differences in root traits whether they grew in stable or in unstable conditions (except *J. curcas* which was not present on the unstable hotspot). *F. tikoua* was more abundant on the unstable hostpot than on the stable site. *P. stricta* and *R. chinensis* both possessed stronger roots in unstable soil conditions. *A. codonocephala* had a higher RAR on the unstable site, but was not as abundant at the unstable hotspot, thus human action may be needed to enhance colonization. Roots of *B. championii*, *A. americana, P. stricta* and *R. chinensis* at the unstable hotspot had higher N content, suggesting that these roots had greater metabolic activity. These species may be more suitable for growing on a site undergoing restoration or a site requiring rapid protection.

### Where is the most efficient location for species on hotspots?

**Figure 12:**
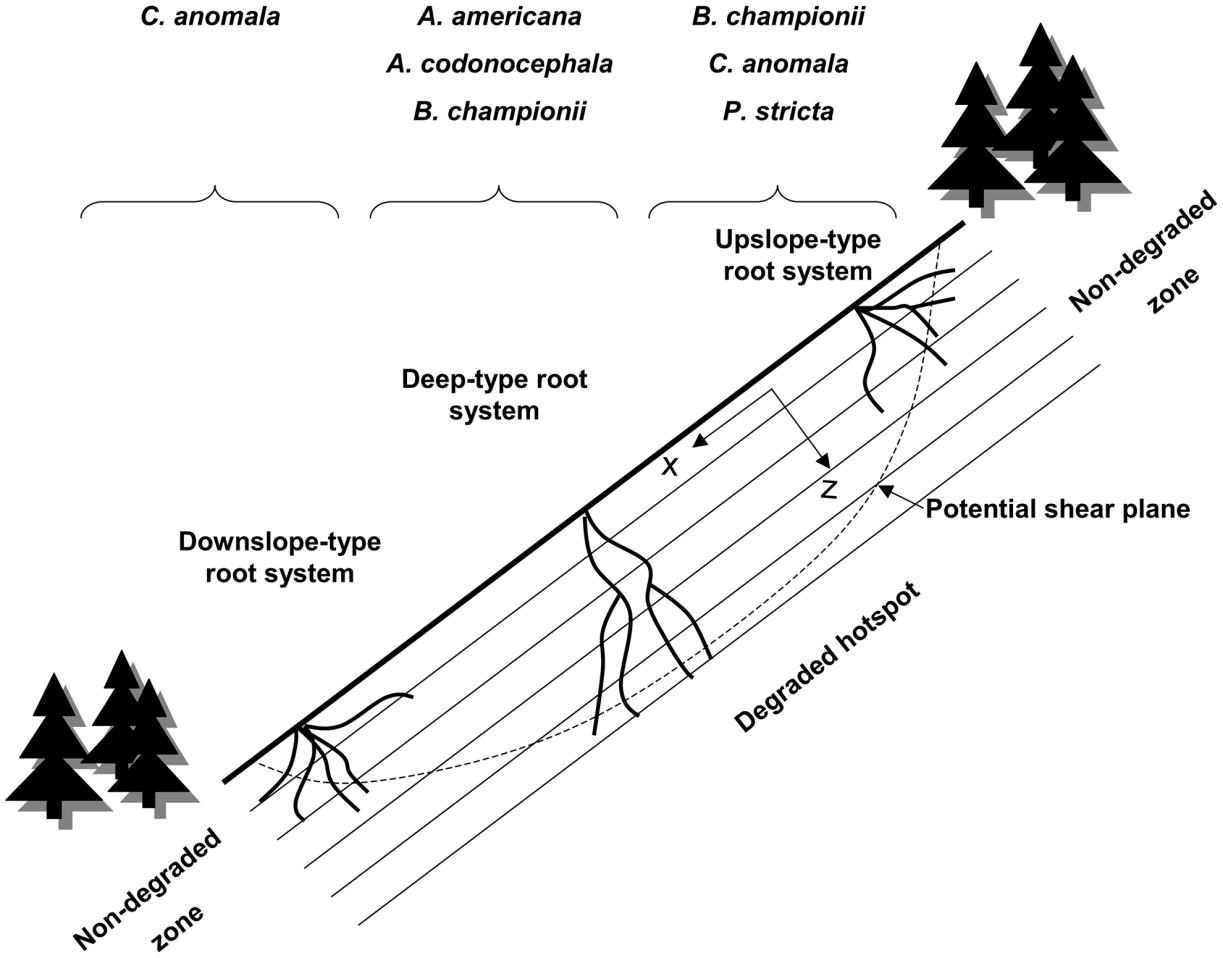
Best species depending on the location on the hotspot of instability. On degraded hotspots, the diversity of root systems will play an important role for slope stability. Roots growing upslope from the stem will have more chance to cross the potential shear plane if the plant grows at the top of the slope. Thus, root systems with desirable traits upslope of the stem will act more efficiently if they are located at the top of the slope, whereas the inverse is applicable for downslope roots. Root systems with desirable traits deeper in the soil will act more efficiently in the middle of the hotspot.

To reinforce a slope against landslides, roots have to cross the potential shear plane. The potential shear plane of a slope can be circular or parallel to the soil surface (Figure 12). Therefore, as it is unlikely that any one species can possess an entire suite of traits that are optimal for increasing slope stability, different species can be planted at different positions along a slope, to optimise soil reinforcement. For example, species with vertical and strong roots will fix soil better in the middle of the slope, whereas plants with more and stronger roots upslope or downslope will better reinforce the top or toe of the slope, respectively (Figure 12), [28]. In our study on small areas of soil slippage, or hotspots, the precise location of species is all the more relevant because the depth of the potential shear zone may increase or decrease rapidly from the top to the bottom of the hotspot. Except for *A. hispidus, F. tikoua* and *J. curcas*, all species had roots which possessed different traits, depending on the depth of the root as well as its orientation with regard to slope direction.

*A. americana* and *B. championii* both had a high density of roots deeper in the soil and roots of the latter species were significantly stronger than shallower roots. Deeper roots of *A. codonocephala* had significantly smaller values of strain. Therefore these three species are able to increase the reinforcement of the potential shear zone at depth.

The root system of *C. anomala* occupied a larger soil volume near the soil surface. The cellulose content in roots of *P. stricta* was higher at surface. Therefore these two species would be more appropriate at the top and at the toe of the landslide rather than in the middle.

To select between top and toe, root systems of *B. championii* and *P. stricta* occupied a greater soil volume deeper in the soil upslope compared to downslope. *P. stricta* had more numerous and stronger roots upslope thus increasing lateral root reinforcement [18]. These two species will therefore be more efficient for improving slope stability when planted at the top of a hotspot (Figure 12).

### Conclusions

In this study, we demonstrated that it is unlikely that any one species possesses an entire suite of root traits necessary to efficiently stabilise a slope with regard to shallow landslides. We suggest the use of mixtures of species, as well as a targeted spatial use of species in particularly fragile hotspots. Such mixtures have been shown to be more efficient at decreasing soil erodibility with regard to water erosion [44], [71], but to our knowledge, this is the first study whereby an in-depth study of desirable root traits with regard to slope stability has been performed. As this was a short-term study, it is now necessary to project root traits' efficacy over time, especially as the woody species we examined were in the juvenile stage.

## Supporting Information

Species List S1 DOCX
