# Supplementary material for: A Framework for Identifying Plant Species to Be Used as ‘Ecological Engineers’ for Fixing Soil on Unstable Slopes
Source: PLoS One. 2014 Aug 8;9(8):e95876. doi: 10.1371/journal.pone.0095876 (PMC4126646; doi:10.1371/journal.pone.0095876)
Supplement: Species List S1 — DOCX [file pone.0095876.s001.docx]

***Supplementary material online***

Functional traits and ethnobotanical uses of species observed at the field site.

In brackets: number of the reference in literature, “obs”: trait deduced from our own observations *in situ* or from discussions with local inhabitants, light grey cells: useful properties, dark grey cells: unsuitable properties.

***List of references:*** (1) Dwivedi et al. 2008; (2) http://plants.jstor.org/upwta/1_128; (3) http://www.globinmed.com/index.php?option=com_content&view=article&id=79083:achyranthes-aspera-linn-amaranthaceae-alt-alternanthera&catid=703:a; (4) Cha et al. 2010; (5) http://plants.jstor.org/upwta/1_11; (6) http://www.hear.org/gcw/species/artemisia_codonocephala/; NB: invasive in Lituany; (7) http://en.wikipedia.org/wiki/Artemisia_%28plant%29; (5) Hartwell 1970; (8) http://www.biodiversityexplorer.org/plants/acanthaceae/barleria.htm; (9) http://en.wikipedia.org/wiki/Bauhinia; (10) http://www.issg.org/database/species/ecology.asp?si=1431&fr=1&sts=&lang=EN; (11) Grubben 2004; (12) Carlquist 1966; (13) Bosu & Apetorgbor 2007; (14) Dweck 2005; (15) Stuart 2006; (16) Hu 2005; (17) Tang et al. 2007; (18) Tang et al. 2009; (19) National Research Council 2006; (20) Carter et al. 2005; (21) Wiart et al. 2004; (22) Galinato et al. 1999; (23) Yuan et al. 2007; (24) Joshi et al. 2001; (25) Wei et al. 1998; (26) Fryer & Hylmö 2009; (27) http://www.efloras.org/florataxon.aspx?flora_id=610&taxon_id=109693; (28) Little & Skolmen 1989; (29) Rojas et al. 1996; (30) Williams & Buxton 1989; (31) West & Noble 1984; (32) Lamb 1981; (33) Jain 1994; (34) Nguyen et al. 1997; (35) Dodd 1961; (36) Howard & Bornstein 1989; (37) Liogier 1995; (38) M’Boob 1991; (39) Fabjan et al. 2003; (40) Anderson 1986; (41) Amatya 1989; (42) Schultes 1985; (43) Invasive Plants of Asian Origin Established in the US and Their Natural Enemies; (44) Kunkel 1984; (45) http://en.wikipedia.org/wiki/Indigo_dye; (46) Polunin & Stainton 1997; (47) Running 2002; (48) Heller 1996; (49) http://en.wikipedia.org/wiki/Lagerstroemia_speciosa; (50) http://www.efloras.org/florataxon.aspx?flora_id=3&taxon_id=200006536; (51) Motooka 2003; (52) Stone 1970; (53) Invasive Plants of Asian Origin Established in the US and Their Natural Enemies http://wiki.bugwood.org/uploads/Paederia.pdf; (54) Demirci et al. 2008; (55) Jansen 2005; (56) Chen 1982; (57) Zhou et al. 1998; (58) Zhao et al. 2005; (59) Chuakul 2000; (60) Mitich 2000, NB: invasive in North America and Central Europe; (61) Li et al. 2000; (62) Hou et al. 2002; (63) Xu et al. 2008; (64) Wang & Gao 2006; (65) Djakpo & Yao 2010; (66) http://en.wikipedia.org/wiki/Castor_oil_plant; (67) http://www.eol.org/pages/2896046; (68) Tian et al. 2006; (69) http://www.friendsoflanecovenationalpark.org.au/Flowering/Flowers/Sigesbeckia_Orientalis.htm. Invasions reported in Australia and La Réunion island; (70) Chen 2000; (71) Roe 1968; (72) Wang et al. 2008; (73) http://www.ars-grin.gov/~sbmljw/cgi-bin/taxon.pl?101867; (74) Flora of China 2003; (75) Mazumder et al. 2001; (76) http://www.bpi.da.gov.ph/Publications/mp/html/d/dalupang.htm; (77) http://www.fs.fed.us/global/iitf/pdf/shrubs/Urena%20lobata.pdf. Invasions reported in North America and Africa; (78) Tan 2006; (79) http://www.invasive.org/browse/subinfo.cfm?sub=6592, NB: invasive in Florida; (80) http://www.efloras.org/florataxon.aspx?flora_id=2&taxon_id=200019509; (81) Wang 2006; (82) http://plants.usda.gov/java/profile?symbol=ARHI3, NB: invasive in Tennessee; (83) Brown & Schultz 1991; (84) Irish 2000

***References for Supplementary material online***

Amatya, S.M. 1989. Variation of *Ficus semicordata* Buch. Ham. Ex Smith Sensu Lato, its taxonomy, distribution and use as a fodder tree in Nepal. Master of Science thesis. Oxford University.

Anderson, E. F. 1986. Ethnobotany of hill tribes of Northern Thailand. II. Lahu medicinal plants. Economic Botany 40: 442-450.

Bosu, P. P., and M. M. Apetorgbor. 2007. *Broussonetia papyrifera* in Ghana: Its invasiveness, impact and control attempts. In: Proceedings of Executive Committee. Management of the Forest Invasive Species Network for Africa (FISNA), 5 pp. Fao Forestry Paper series. Pietermaritzburg, South Africa, May 16.

Brown, L. E., and J. Schultz. 1991. *Arthraxon hispidus* (Poaceae), new to Texas. Phytologia 71: 379-381.

Carlquist, S. 1966.  The biota of long-distance dispersal. II. Loss of dispersibility in Pacific Compositae. Evolution 20: 30-48. doi:10.2307/2406147.

Carter, C. T., C. M. Grieve, J. A. Poss, and D. L. Suarez. 2005.  Production and ion uptake of *Celosia argentea* irrigated with saline waste waters. Scientia Horticulturae 106: 381-394. doi:10.1016/j.scienta.2005.04.007.

Cha, J. D., Y. H. Kim, and J. Y. Kim. 2010. Essential oil and 1,8-cineole from *Artemisia lavandulaefolia* induces apoptosis in KB cells via mitochondrial stress and caspase activation. Food science and biotechnology 19: 185-191.

Chen, C. J. 1982. A monograph of *Pilea* (Urticaceae) in China. Bulletin of Botanical Research, Harbin 2: 31-132.

Chen, X. Q. 2000. *Smilax linnaeus*, Sp. Flora of China 24: 96-115.

Chuakul, W. 2000. Medicinal plants in Khao Kho district, Phetchabun Province, Thailand. Pharmaceutical biology 38: 61-67.

Demirci, F., K. Guven, B. Demirci, M.Y. Dadandi, and K.H.C. Baser. 2008. Antibacterial activity of two *Phlomis* essential oils against food pathogens. Food Control 19: 1159-1164.

Djakpo, O., and W. Yao. 2010. *Rhus chinensis* and *Galla chinensis* – folklore to modern evidence: review. Phytotherapy Research 24: 1739-1747. doi:10.1002/ptr.3215.

Dodd, A. P. 1961. Biological control of *Eupatorium adenophorum* in Queensland. Australian Journal of Science 23: 356-65.

Dweck, A. C. 2005. A review of the Paper Mulberry (*Broussonetia papyrifera*) (L.) Hert. ex Vent.

http://www.rarefruit.org/PDF_files/Broussonetia_papyrifer.pdf.

Dwivedi, S., R. Dubey, and K. Mehta. 2008. *Achyranthes aspera* Linn. (Chirchira): A magic herb in folk medicine. Ethnobotanical Leaflets 12: 670-676.

Fabjan, N., J. Rode, I. J. Košir, Z. Wang, Z. Zhang, and I. Kreft. 2003. Tartary Buckwheat (*Fagopyrum tataricum* Gaertn.) as a source of dietary rutin and quercitrin. Journal of Agricultural and Food Chemistry 51: 6452-6455. doi:10.1021/jf034543e.

Fryer, J., and B. Hylmö. 2009. Cotoneasters: a comprehensive guide to shrubs for flowers, fruit, and foliage. Timber Press. Portland, Oregon.

Galinato, M. I., K. Moody, and C. M. Piggin. 1999. Upland rice weeds of south and Southeast Asia. International Rice Research Institute.

Grubben, G. J. H. 2004. Vegetables. PROTA.

Hartwell, J.L. 1970. Plants used against cancer—A survey. Lloydia 33: 288-392.

Heller, J 1996. Physic nut, *Jatropha curcas* L. International Plant Genetic Resources Institute, Institut für Pflanzengenetik und Kulturpflanzenforschung Gatersleben, Germany. Bioversity International.

Hou, J. J., W. K. Wei, H. Xue, and H. Zhang. 2002. Advances on the research of *Pyracantha Fortuneana* (Maxim.) Li and its products. Journal of Hubei Institute for Nationalities.

Howard, R. A., and A. J. Bornstein. 1989. Flora of the Lesser Antilles: Leeward and windward islands. Arnold Arboretum, Harvard University.

Hu, S. Y. 2005. Food plants of China. Chinese University Press.

Irish, G. 2000. Agaves, yuccas, and related plants: A gardener’s guide. Timber Press. Portland, Oregon.

Jain, R. K. 1994.  Fuelwood characteristics of medium tree and shrub species of India. Bioresource Technology 47: 81-84. doi:10.1016/0960-8524(94)90032-9.

Jansen, P. C. M. 2005. *Phyllanthus emblica* L. In Jansen, P.C.M. & Cardon, D, editors. Plant Resources of Tropical Africa (PROTA). Dyes and tannins/Colorants et tanins. Tome 3. Wageningen, The Netherlands.

Joshi, B., S. P. Singh, Y. S. Rawat, and G. Deepti. 2001. Facilitative effect of *Coriaria nepalensis* on species diversity and growth of herbs on severely eroded hill slopes. Current Science 80: 678-682.

Lamb, S. H. 1981. Native trees and shrubs of the Hawaiian islands. 1^st^ edition. Sunstone Press, Santa Fe, New Mexico.

Li, N., Z. D. Min, and M. W. Hou. 2000. Two new oleanene-type triterpenoid saponins from *Pueraria peduncularis*. Chinese Chemical Letters 11: 343-346.

Liogier, A. H. 1995. Descriptive flora of Puerto Rico and adjacent islands: Spermatophyta - Dicotyledoneae. Melastomataceae to Lentibulariaceae. La Editorial, UPR.

Little, E., and R. G. Skolmen. 1989. Common forest trees of Hawaii (native and introduced). USDA, Forest Service, Agricultural Handbook: 321pp.

M’Boob, S. S. 1991. Preliminary results of a survey and assessment of *Chromolaena odorata* (Siam weed) in Africa. Biotropical Special Publication: 51-55.

Mazumder, U. K., M. Gupta, L. Manikandan, and S. Bhattacharya. 2001. Antibacterial activity of *Urena lobata* root. Fitoterapia 72: 927-929. doi:10.1016/S0367-326X(01)00330-6.

Mitich, L. W. 2000. Kudzu [*Pueraria lobata* (Willd.) Ohwi]. Weed Technology 14: 231-235.

Motooka, P. S. 2003. Weeds of Hawaii’s pastures and natural areas: An identification and management guide. College of Tropical Agriculture and Human Resources, University of Hawaii, Mānoa.

National Research Council. 2006. Lost crops of Africa. Volume II: Vegetables. The National Academies Press, Washington, D.C.

Nguyen, T. T., L. A. Ha, and J. Casanova. 1997. Elsholtzia sauvage (*Elsholtzia winitianna* Craib.) : Une drogue à huile essentielle riche en cinéol. *Elsholtzia winitianna* Craib: A medicinal plant with volatile oil rich in cineol. Revue pharmaceutique: 40-41.

Polunin, O., and A. Stainton. 1997. Flowers of the Himalaya. Oxford University Press.

Roe, K. E. 1968. *Solanum verbascifolium* L., misidentification and misapplication. Taxon 17: 176-179. doi:10.2307/1216510.

Rojas, A., S. Cruz, H. Ponce-Monter, and R. Mata. 1996. Smooth muscle relaxing compounds from *Dodonaea viscosa* . Planta Medica 62: 154-159. doi:10.1055/s-2006-957840.

Schultes, R. E. 1985. Plants for human consumption. Economic Botany 39: 176-176. doi:10.1007/BF02907842.

Stone, B. C. 1970. The flora of Guam. Micronesica 6: 1-659.

Stuart, D. D. 2006. Buddlejas. Timber Press, Portland, Oregon.

Tan, X. F. 2006. Status and suggestion on development of *Vernicia fordii*. Nonwood Forest Research.

Tang, C., B. Shi, W. Gao, F. Chen, and Y. Cai. 2007. Strength and mechanical behavior of short polypropylene fibre reinforced and cement stabilized clayey soil. Geotextiles and Geomembranes 25: 194-202. doi:10.1016/j.geotexmem.2006.11.002.

Tang, C. Q., M. H. Zhao, X. S. Li, M. Ohsawa, and X. K. Ou. 2009. Secondary succession of plant communities in a subtropical mountainous region of SW China. Ecological Research 25: 149-161. doi:10.1007/s11284-009-0644-z.

Tian, X. Y., Y. H. Wang, Q. Y. Yang, X. Liu, W. S. Fang, and S. S. Yu. 2006. Jacaranone glycosides from *Senecio scandens*. Journal of Asian Natural Products Research 8: 125.

Wang, D. X., P. Liu, Y. H. Chen, D. H. Guo, H. Y. Ren, and M. L. Chen. 2008.  Stimulating effect of catechin, an active component of *Spatholobus suberectus* Dunn, on bioactivity of hematopoietic growth factor. Chinese Medical Journal 121: 752-755.

Wang, R. Z. 2006. The occurrence of C4 photosynthesis in Yunnan province, a tropical region in South-western China. Photosynthetica 44: 286-292. doi:10.1007/s11099-006-0020-y.

Wang, Z., and X. Gao. 2006.  Predating strategy of rodents on acorns of *Quercusaliena* var. *acuteserrata* under different predating risks and fate of acorns. Acta Ecologica Sinica 26: 3533-3540. doi:10.1016/S1872-2032(06)60052-8.

Wei, H., F. Zeng, M. Lu, and R. Tang. 1998.  Studies on chemical constituents from the root of *Coriaria nepalensis* Wall (*Coriaria sinica* Maxim). Yao Xue Xue Bao: Acta Pharmaceutica Sinica 33: 688-692.

West, J. G., and I. R. Noble. 1984. Analyses of digitised leaf images of the *Dodonaea viscosa* complex in Australia. Taxon 33: 595-613. doi:10.2307/1220777.

Wiart, C., S. Mogana, S. Khalifah, M. Mahan, S. Ismail, M. Buckle, A. K. Narayana, and M. Sulaiman. 2004. Antimicrobial screening of plants used for traditional medicine in the state of Perak, Peninsular Malaysia. Fitoterapia 75: 68-73. doi:10.1016/j.fitote.2003.07.013.

Williams, P.A., and R.P. Buxton. 1989. Response to reduced irradiance of 15 species of native and adventive shrub and tree seedlings from Eastern Canterbury. New Zealand Journal of Ecology 12: 95-101.

Xu, F., W. H. Guo, and R. Q. Wang. 2008. Habitat effects on leaf morphological plasticity in *Quercus acutissima*. Acta biological Cracoviensia 50: 19-26.

Yuan, X. H., B. G. Li, C. X. Xu, M. Zhou, H. Y. Qi, and G. L. Zhang. 2007. Three new limonoids from *Cipadessa cinerascens*. Chemical & Pharmaceutical Bulletin 55: 902-904.

Zhao, X., H. Sun, A. Hou, Q. Zhao, T. Wei, and W. Xin. 2005. Antioxidant properties of two gallotannins isolated from the leaves of *Pistacia weinmannifolia*. Biochimica et Biophysica Acta (BBA) - General Subjects 1725: 103-110. doi:10.1016/j.bbagen.2005.04.015.

Zhou, Y., D. Watts, Y. Li, and X. Cheng. 1998. A case study of effect of lateral roots of *Pinus yunnanensis* on shallow soil reinforcement. Forest Ecology and Management 103: 107-120. doi:10.1016/S0378-1127(97)00216-8.
